# Applications of 3D Bioprinting Technology to Brain Cells and Brain Tumor Models: Special Emphasis to Glioblastoma

**DOI:** 10.1021/acsbiomaterials.3c01569

**Published:** 2024-04-26

**Authors:** Ilkay Irem Ozbek, Hale Saybasili, Kutlu O. Ulgen

**Affiliations:** † Department of Chemical Engineering, Bogazici University, Istanbul 34342, Turkey; ‡ Institute of Biomedical Engineering, Bogazici University, Istanbul 34684, Turkey

**Keywords:** tumor microenvironment, bioinks, 3D in vitro models, drug screening

## Abstract

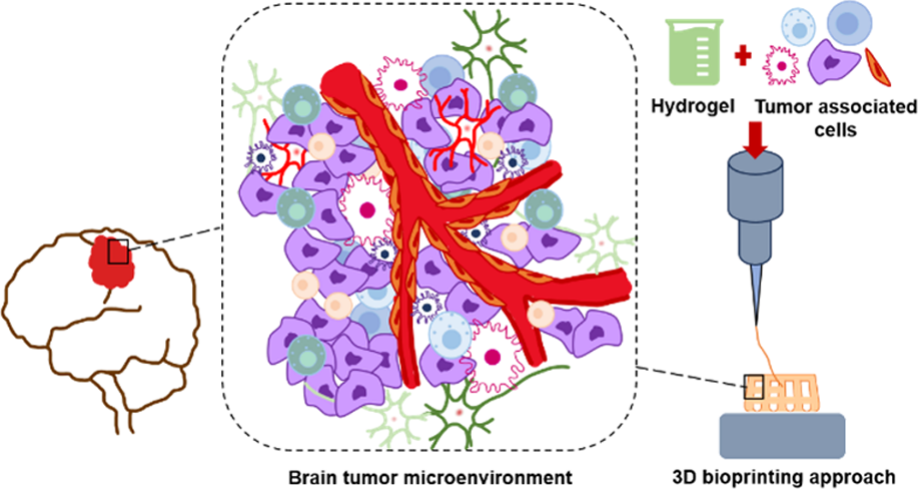

Primary brain tumor is one of the most fatal diseases. The most malignant type among them, glioblastoma (GBM), has low survival rates. Standard treatments reduce the life quality of patients due to serious side effects. Tumor aggressiveness and the unique structure of the brain render the removal of tumors and the development of new therapies challenging. To elucidate the characteristics of brain tumors and examine their response to drugs, realistic systems that mimic the tumor environment and cellular crosstalk are desperately needed. In the past decade, 3D GBM models have been presented as excellent platforms as they allowed the investigation of the phenotypes of GBM and testing innovative therapeutic strategies. In that scope, 3D bioprinting technology offers utilities such as fabricating realistic 3D bioprinted structures in a layer-by-layer manner and precisely controlled deposition of materials and cells, and they can be integrated with other technologies like the microfluidics approach. This Review covers studies that investigated 3D bioprinted brain tumor models, especially GBM using 3D bioprinting techniques and essential parameters that affect the result and quality of the study like frequently used cells, the type and physical characteristics of hydrogel, bioprinting conditions, cross-linking methods, and characterization techniques.

## 1 Introduction

Brain tumors are life-threatening diseases that affect people, especially children, detrimentally. Glioblastoma (GBM) is the most malignant type of brain tumor, and it causes the death of more than 65% of patients in 2 years following diagnosis.^1^

Apart from the epigenetic/genetic variations occurring in tumor cells, the remodeling of components in the tumor microenvironment (TME) is a main criterion that affects tumor progression.^2^ It was suggested that tumors should be referred to as small organs where malignant cells interact with each other and their microenvironment.^3^

The TME contains endothelial cells, fibroblasts, and immune cells and also extracellular matrix components such as collagen, hyaluronan, laminin, and fibronectin.^2,4^ In spite of recent advancements, modeling TME is a huge challenge when it comes to dealing with cancer models.

2D models that reflect the tumor environment have been employed for decades in the investigation of tumor progression and testing drugs. However, these conventional models do not reflect the tumor environment accurately, and receptor and signaling molecules were indicated to be decreased or lost in cells cultured in 2D models.^5,6^ Although animal models provide important clues about cancer biology, they have downsides such as being biologically different from humans and having high cost and ethical dilemmas.^5^ Recently, 3D in vitro models have been presented as alternatives that bridge the gap between two methods to fulfill the disadvantages and shortcomings of animal models and 2D models.

3D bioprinting technology, one of the recent tissue engineering applications, allows researchers to make progress in cancer tissue modeling with the encapsulation of cells in biomaterials that mimic matrix characteristics and maintain cell viability. This technology enables scalable and relatively rapid manufacture, excellent versatility in cell positioning, and layer-by-layer deposition of biological and chemical components with reproducibility.^7,8^ While, in 2D models, cells can only attach and proliferate on flat surfaces, which does not recapitulate in vivo cell morphology resulting in poor cell communication, in 3D models, cells can grow in any direction without contacting the surface, leading to better cell–cell and cell–ECM interactions; thus, these models better reflect an in vivo environment.^9^ Furthermore, it is possible to fabricate a complex vascular network that delivers nutrients, oxygen, and signaling components to malignant cells using the 3D bioprinting approach.^10^ Designing personalized models renders this technology desirable, especially for malignancies like GBM where cells demonstrate different characteristics throughout tumor regions and among patients.^11^

While most papers have focused on 3D bioprinted cancer models,^5,12,13^ 3D bioprinted neural tissues/brain models,^14−20^ and neurodegenerative diseases,^21^ this Review specifically covers 3D bioprinted brain tumor models that reflect TME by utilizing different cell types, extracellular matrix components, and their use in drug screening. Additionally, 3D bioprinted models containing neuroblastoma (NB) cell lines were covered as NB cell lines have the potential to differentiate into neurons; thus, they can be utilized in both 3D neural tissue and brain tumor models. The frequently used hydrogels and their physical properties, cell types, bioprinting conditions, and cross-linking methods that influence cell viability and stability of structure are discussed below. The currently used characterization techniques that illustrate cell metabolism, viability, interaction with each other, and morphology are also elaborated.

We hope that this study will guide those who want to be informed about current studies where brain tumor models are developed using bioprinting means.

## 2 Key Parameters

### 2.1 Cells in 3D Bioprinting

Cell selection is a significant step when constructing 3D models in which tumor cell lines, primary patient cells, and tumor stem cells (SCs) are generally used.^5,15^ Tumor cell lines are the most prevalently utilized cells in studies; however, the phenotypic alterations they have gone through make them unfavorable for research.^5^ Key parameters including cell types used for 3D bioprinted GBM models are shown in Table 1. Various studies harnessed the U-87 MG GBM cell line to construct a 3D bioprinted GBM model.^6,22−27^ This cell line, which was acquired from a male patient with GBM, is widely used as it can be obtained and handled easily.^28^ However, the U-87 MG GBM cell line was demonstrated to possess a different genetic profile from GBM.^28^ Other widely employed cell lines are U118-MG^29−32^ and U251-MG.^33,34^

**Table 1:** Bioprinting Parameters for Glioblastoma Tumor Models^a^

application	cell type	bioprinting technique	bioink type	cross-link method	viability technique	reference
3D bioprinted GBM model	GBM cells (U87MG)	Extrusion bioprinter	Fibrin, alginate, genipin	CaCl_2_, chitosan, thrombin	Live/dead assay kit, fluorescence microscope	(25)
3D bioprinted GBM fiber model	Glioma SCs (GSC23), MSC	Coaxial extrusion bioprinter	Alginate, gelatin, fibrinogen	CaCl_2_, thrombin	Live/dead assay kit, fluorescence microscope	(45)
3D bioprinted GBM model	GBM cells (U118)	Multinozzle extrusion bioprinter	Sodium alginate, gelatin, and fibrinogen	CaCl_2_, thrombin	Live/dead assay kit, fluorescence microscope	(31)
3D bioprinted GBM organoid model	Human liver cancer cells (HepG2), human colorectal cancer epithelial cells (Caco2), patient derived GBM cells	Extrusion bioprinter with immersion bioprinting technique	Coll-MA, HA, HyStem–HP hydrogel	Photo-induced cross-linking	Live/dead assay kit, fluorescence microscope	(38)
3D bioprinted GBM model	Glioma SCs (SU3), MSCs	Coaxial extrusion bioprinter, extrusion bioprinter	Sodium alginate, gelatin, fibrinogen	CaCl_2_, thrombin	Live/dead assay kit, fluorescence microscopy	(46)

a Abbreviations: glioblastoma, GBM; stem cell, SC; mesenchymal stem cells, MSCs; methacrylated type 1 collagen, Coll-MA; hyaluronic acid, HA.

Primary cells that are acquired from patients have psychological and biological properties found in tumor cells; therefore, they reflect most accurately the real situation in patients and they are generally employed to create personalized tumor models.^5^ Many studies use patient-derived GBM cell lines to develop their models.^35−38^

The fact that SCs can be differentiated into other cells renders them desirable in tissue engineering applications.^15^ SCs contribute to the construction of a much more realistic tumor environment in 3D bioprinted models since self-renewal features and indefinite replication of them are directly associated with tumor viability and migration.^5^ There are several types of SCs which are human embryonic stem cells (hESCs), mesenchymal stem cells (MSCs), and human induced pluripotent stem cells (hiPSCs).^16^

hESCs are obtained from embryos while MSCs are multipotent stromal cells that can be converted to myocytes, osteoblasts, adipocytes, and chondrocytes. MSC cells, SH-SY5Y, and human primary umbilical vein endothelial cells (HUVECs) were used to prepare three different bioinks and construct the 3D bioprinted NB model where these cells make up stroma, rosettes, and vasculature parts of the model, respectively.^39^ MSC cells were found to stimulate the formation of the elastic matrix which is a hallmark of NB.^39^

Neural stem cells (NSCs) can be developed into neurons, astrocytes, and oligodendrocytes.^16^ Like hESCs, human induced pluripotent stem cells (hiPSCs) can be transformed into almost any cell in the nervous system. hiPSCs have been employed in 3D models of brain injury^40^ and neurodegenerative diseases including Parkinson’s disease,^41^ Alzheimer’s disease,^42^ and Amyotrophic lateral sclerosis.^43^ Additionally, the use of hiPSCs is promising in terms of taking a step into the field of personal medicine.^18^ Fantini et al. employed adult somatic cells that can be differentiated into iPSCs and then into NSCs by using iPSC technology to create neural organoid originating from patients’ cells.^44^

The utilization of myriad cell types such as endothelial and fibroblast in 3D bioprinted tumor models ensures better recapitulation of TME composed of blood vessels, stromal cells, pericytes, and immune cells.^12^ Fibroblasts promote angiogenesis by excreting angiogenic factors like vascular endothelial growth factor (VEGF) and thus lead to the formation of new vessels that support malignant cells with necessary nutrients.^23^ A blood vessel layer was created by incorporating lung fibroblasts and HUVECs into a hydrogel blend. Once blood vessel formation was observed, multicellular tumor spheroids containing GBM cells (U87 MG) were placed on the aforementioned layer and kept until HUVECs disseminated to spheroids and caused angiogenesis formation as malignant cells moved to the layer. As a result, temozolomide (TMZ) and the angiogenic inhibitor sunitinib responded similarly in the 3D bioprinted construct to that observed in real tumor tissue, suggesting that the proposed TME model is an efficient platform for drug testing.^23^

The tumor-immune microenvironment (TIME) includes natural killer cells, neutrophils, dendritic cells, macrophages, B cells, and T cells.^3^ The TME of most primary gliomas has the most abundant microglia and glioma-associated macrophages related to the central nervous system (CNS).^47^ Immune cells including monocytes and macrophages were used in several studies.^3,24,48,49^ A 3D bioprinted GBM model was created using U-87 MG GBM cell lines with a macrophage-like Mono-Mac-6 (MM6) cell line and fibroblasts to better recapitulate TME.^24^ In a separate model, they employed three different glioma stem cells (GSCs) (G144, G166, and G7), microglia, and glioma-associated stromal cells obtained from patients.

The inclusion of MM6 into bioink reduced the sensitivity of the cisplatin. GSCs also demonstrated increased resistance to TMZ and cisplatin relative to 2D cell cultures suggesting a promising practice for preclinical drug tests.^24^

Apart from neurons, glial cells which are Schwann cells, oligodendrocytes, astrocytes cells, microglia, and ependymal are found profusely in the brain. Several studies have utilized Schwann cells since stromal cells including Schwann cells are found in stromal tumors like NB.^50,51^ Many studies also utilized the NB cell lines (e.g., SH-SY5Y) which can be differentiated into neural cells to create 3D bioprinted neural tissue or NB models.^50−53^

Neural differentiation has been carried out using various procedures including applications of alltrans-retinoic acid (vitamin A derivative), nerve growth factor (NGF), oestradiol, 12-*O*-tetradecanoyl phorbol 13-acetate, cholesterol, and brain-derived neurotrophic factor (BDNF).^54^ The parameters of 3D bioprinted tumor models including NB cells are shown in Table 2.

**Table 2:** Bioprinting Parameters for Tumor Models with Neuroblastoma Cells^a^

application	cell type	bioprinting technique	bioink type	cross-link method	viability technique	reference
3D bioprinted NB model	NB cells (SK-N-BE (2)), NB cells (SH-SY5Y), SW10 mouse Schwann cells	Extrusion bioprinter	AlgMA, GelMA	Photo-induced cross-linking		(51)
3D bioprinted vascularized NB model	Patient derived NB cells (IMR5), HUVECS	Extrusion bioprinter (embedded bioprinting method)	GelMA, Carbopol as supporting bath material	Photo-induced cross-linking	Confocal microscopy, live/dead viability assay kit, AB assay	(57)
3D bioprinted NB model	NB cells (IMR-32), HEK cells (HEK293, CRL-1573)	Multinozzle extrusion bioprinter	collagen, sodium alginate, gelatin	CaCl_2_	XTT assay	(58)
3D bioprinted neural model	Neuro2a mouse NB cells	Extrusion bioprinter	NOCC, agarose	CaCl_2_	Live/dead assay kit, confocal microscope	(59)

a Abbreviations: neuroblastoma, NB; methacrylated alginate, AlgMA; gelatin methacryloyl, GelMA; human umbilical vein cells, HUVECS; Alamar Blue, AB; human embryonic kidney cells, HEK cells; *N*,*O*-carboxymethyl chitosan, NOCC.

### 2.2 Bioprinting Methods

Neural tissues can be created utilizing various types of bioprinting methods which are extrusion-based bioprinting (EBB), light-based bioprinting, and droplet-based (inkjet) bioprinting.^11,55^ Extrusion bioprinting is the most commonly employed bioprinting method as it has an affordable cost, can be easily installed, and allows one to design constructs using desired bioinks with high density.^3,12^ EBB creates 3D constructs by discharging small quantities of bioinks by sheer force through a nozzle or multinozzle. The mechanical means used in the extrusion process can be categorized as pneumatic-based, screw-driven, and piston-driven systems.^56^ Widely used pneumatic-based bioprinting can be controlled easily by altering the pressure. However, delays in controlling pressure might lead to a decrease in precision in the spatial control of discharged bioinks.^11^

The mechanical-based system offers better control in the deposition of bioinks than the pneumatic-based system.^56^ The screw-driven approach is particularly preferred with viscous materials.^11^ Another important advantage of the extrusion technique is that it can be used with hydrogels containing high cell density.^12^ However, cell death might be observed throughout bioprinting under high shear stresses.

Coaxial extrusion bioprinting is an advanced extrusion technique where two or more bioinks are dispensed simultaneously with a coaxial nozzle.^60^ Several studies used coaxial extrusion bioprinters to be able to construct core–shell hydrogel microfibers.^30,45^ Dai et al. used hydrogel and cell suspension as shell and core, respectively, to better reflect TME.^45^ The same group employed the same technique to model core–shell hydrogel microfibers where they concurrently bioprinted GSCs and glioma cell lines from the outer and central exits of a nozzle, respectively.^30^

**Figure 1:**
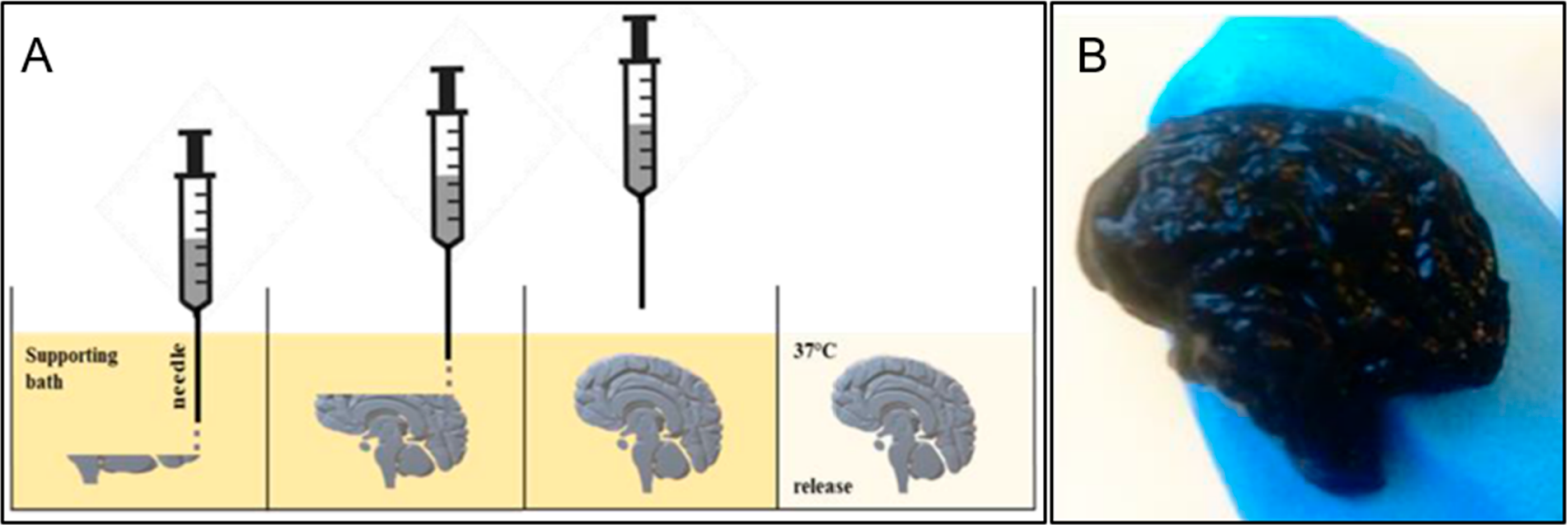
(A) The construction process of the 3D bioprinted model using the FRESH bioprinting technique. (B) Brain-shaped scaffold constructed using cellulose-based bioink. Reproduced with permission from ref (65). Open Access.

The main challenge in bioprinting is the deterioration of structures made of soft bioinks because of gravity and a decrease in bioprinting fidelity.^61^ Layer-by-layer bioprinting of most bioinks is very difficult without support, and they do not cure rapidly and with enough stiffness to ensure for structure integrity throughout bioprinting. Innovative methods employing physical media to support embedded bioinks during bioprinting have recently surfaced, resulting in the creation of 3D models that more accurately replicate the intricate form of the human body.^62^ Embedded bioprinting can be utilized with low viscous or mechanically weak bioinks such as gelatin, collagen, and alginate to produce intricate structures with less geometric limitations and higher resolution.^62,63^ Several NB/neural models were constructed by using a gelatin or Carbopol bath as a physical support.^57,64,65^ Freeform Reversible Embedding of Suspended Hydrogels (FRESH), a well-known EBB technique, usually demonstrates yield stress, ensuring freeform bioprinting and its liquid part is compatible with cross-linking mechanisms of various bioinks including fibrinogen, collagen, alginate, decellularized extracellular matrix, and HA.^62,66^ Bordoni et al. employed FRESH to develop a realistic brain scaffold (Figure 1).^65^ Once the construct was developed using cellulose-based bioink with a gelatin-supporting bath, it was placed at 37 °C for 60 min. This technique proved itself to be beneficial when it comes to constructing structures with fine details.^65^

Droplet-based bioprinting employs several types of energy sources including thermal, system, or piezoelectric actuators to form bioink droplets.^12^ This approach ejects small amounts of bioink precisely for better resolution than most bioprinting methods.^67^ This strategy enables biocompatible and fast production which is advantageous for maintaining the viability of the cells.^18^ However, bioink might be subject to mechanical stress, which can be harmful to the cells it contains. Additionally, the nozzles can easily clog, so biomaterials with low viscosities should be used.^14^

Light-assisted bioprinting methods have certain advantages such as high-speed bioprinting with good resolution and maintaining high cell viability even with sensitive cells like SCs as they operates with lower sheer pressures compared to other bioprinting techniques.^11,55^ The most widely employed light-based bioprinting methods are laser-based bioprinting, digital light processing (DLP)-based bioprinting, stereolithography (SLA), computed axial lithography (CAL), and two-photon polymerization (TPP)-based bioprinting.^11^

A laser-based bioprinter has a pulsed laser employed as an energy source, which irradiates a ribbon with energy-absorbing metal leading to evaporation of bioink, and then, it is collected in a droplet form on the receiving substrate. Laser-based bioprinters offer a noncontact, nozzle-free operation with good resolution and maintain cell viability with a wide range of bioink viscosity.^14,68^ However, low flow rates are required, and issues like metallic residue contamination have been observed in this technique.^14^

Stereolithography is a light-assisted bioprinting strategy where cell-laden hydrogel is solidified via photoinduced cross-linking in a layer-by-layer manner regulated by a movable platform along the *z*-axis.^69^ This approach allows the development of complex structures with advantages including high efficiency, rapid bioprinting, and superior scalability as well as high resolution.^70^

Digital light processing (DLP)-based bioprinting is very similar to stereolithography. However, it is a projection-based bioprinting approach, where liquid bioink is converted into solid 3D constructs via inducing photopolymerization by projection light.^71^ DLP is faster than SLA especially when it comes to the development of larger models.^72^ Unlike traditional bioinks, the bioinks used in light-assisted 3D bioprinting are required to be incorporated with photoreactive materials to allow rapid photopolymerization of the bioinks. The insufficiency of photosensitive materials has restricted the use of DLP and SLA.^70^

Computed axial lithography (CAL) allows the production of a 3D construct through a single rotation of bioinks with predetermined pattern projections.^11^ This approach is based on the back-projection algorithm employed in computed tomography reconstruction.^73^ CAL ensures a faster bioprinting process with scalability.^70^

Two-photon polymerization (TPP)-based bioprinting is a light-assisted direct-writing approach that polymerizes bioinks by the concurrent absorption of two photons from a femtosecond laser.^74^ Two-photon lithography is very useful in the development of constructs with small-scale traits for biomedical engineering applications. However, the low speed of this approach renders the production of macro-scale constructs with a good resolution very challenging.^74^

Despite the recent advanced techniques like CAL that decrease bioprinting time significantly, bioprinting human-scale tissues is still a long procedure, rendering it difficult to preserve cell viability. Embedding strategies such as FRESH support cell survival providing a bath containing growth factor and cell media during bioprinting. Still, producing focused solutions to this problem is one of the most important needs of this field.^75^

### 2.3 Hydrogel Properties

#### 2.3.1 Selection of Hydrogel Material

Selecting the right hydrogel with biocompatible and biodegradable multimaterials for each model is very significant to mimic TME of neural tissues and maintain cell viability.^15^ There are three types of biomaterials utilized in hydrogel preparation, natural, semisynthetic, and synthetic biomaterials. Natural biomaterials provide an environment where cells can develop by imitating ECM and ensuring biocompatibility.^18^ The most frequently employed biomaterials in 3D neural tissue applications are hyaluronic acid (HA), gelatin, collagen, chitosan, and alginate.^15^ HA, the most plentiful substance of ECM in the healthy brain, supports and controls GBM development and migration via CD44 and hyaluronan-mediated motility receptors.^49^ HA and gelatin methacryloyl (GelMA) were employed to mimic GBM by using NSCs, patient-derived GSCs, astrocytes, and macrophages.^49^ The expression level of the GSC gene in the 3D bioprinted model where all cells were employed was found to be higher than its counterpart in the spheroids including only tumor cells.^49^

Collagen and Matrigel containing laminin (∼60%), collagen (∼30%), entactin (∼6%), and perlecan (∼2–3%) are promising biomaterials since collagen is one of the main components of ECM.^15^ They also maintain the highest cell viability in neural tissues.

Almost all studies designing 3D bioprinted GBM models utilized natural biomaterials. Almost 85% of these studies modeling GBM have employed collagen or collagen-derived gelatin and/or alginate (Table S1). A natural polymer Gellan Gum (GG), which is produced from the microorganism *Pseudomonas elodea*, possesses similar mechanical strength to that of gelatin at low concentrations.^76^ It also has edges over other hydrogels such as its biocompatibility, low cost, desirable rheological characteristics, and high gelling potential. GG has been combined with RGD peptide and primary neural cells as a bioink to obtain neural tissues.^76^ RGD improves the viability of neural and glial cells and contributes to neural network development. The chitosan–gelatin combination, which is biocompatible and supports adherence of NB cells, displayed high viability with IMR-32 NB cells for 5 days.^77,78^ Alginate obtained from algae is also a desirable hydrogel material as it promotes neural cells and possesses properties such as its biocompatibility and capability to cross-link.^15^ The gelatin–alginate combination has been frequently utilized to model malignant neural tissues (Table S1). In addition to alginate, the hydrogels obtained from plants, algae, and land including nanocellulose, agarose, pectin, fucoidan, carrageenan, and starch are preferred.^79^ NB cells proliferated and maintained viability for 7 days in cellulose–alginate-containing hydrogels.^64^ Silk fibroin (SF), another natural material obtained from silkworms, is also appealing due to its biocompatibility, biodegradability, and mechanical and shear-thinning features,^80^ but natural biomaterials may be structurally inadequate, and unsteady, which renders bioprinting challenging and results in unstable tissue structure for cells.^80^ Additionally, batch-to-batch variation of natural biomaterials remains a challenge. The biological and mechanical characteristics of ECM biomaterials change according to the age of the animals or environmental conditions.

The problem of batch-to-batch variation is much less common with synthetic biomaterials.^75^ Synthetic biomaterials such as Pluronic F127 and polyethylene glycol (PEG) are useful regarding the adjustability of their physical characteristics, absorbability, and hydrophilicity.^5^ However, they show poor biological activity. Combining synthetic materials with natural materials can increase biocompatibility. Graphene oxide (GO) was used in chitosan-based hydrogel to improve electrical conduction and mechanical features.^81^ Among bioinks with different GO concentrations, 0.5 wt % GO considerably enhanced the yield stress, viscosity, and storage modulus of chitosan-based bioinks.

A combination of HA and poly(ethylene glycol) was also employed to encapsulate long-term neuroepithelial stem cells (lt-NESs) and SH-SY5Y cells that were not differentiated or differentiated with retinoic acid (RA) in the presence and absence of laminin.^52^ Differentiated cells encapsulated in hydrogels were viable for 10 days, and they also formed spheroids. It was detected that the presence of laminin did not affect the viability of NB cells while it influenced the viability of lt-NES significantly. Laminin that encloses peripheral nerves and vessels is found in both peripheral and nervous systems.^14^ According to another study where GSCs were embedded within collagen with different laminin concentrations, cells grow more quickly with higher laminin concentrations.^82^

In another work, a tumor heterogenic microenvironment was replicated by using two bioinks.^22^ The thermo-reversible Pluronic F127 was used as a sacrificial bioink along with endothelial cells and pericytes to obtain a vascular lumen in a bioprinted chip. Gelatin and fibrinogen blend containing astrocytes, patient-derived GBM cells, and microglia was utilized for stroma bioink. Different drug responses to TMZ were detected in the 3D bioprinted model with three patient-derived GBM cells, unlike the 2D model where no difference between cells was observed. The 3D bioprinted GBM model was proved to be an efficient preclinical platform by giving similar growth rate, genetic profile, and drug sensitivities to animal models.^22^

In addition, the combination of cellulose nanofibrils and carbon nanotubes which renders scaffolds electrically conductive has been employed specifically for neural tissue development.^83^ Charged cellulose nanofibrils can uniformly distribute carbon nanotubes leading to modeling electrically conductive constructs. Conductive nanotubes improve neural network formation by increasing the electrical interaction of neurons. This novel approach enhanced the proliferation, attachment, and differentiation of SH-SY5Y cells.^83^ Electrostatic forces have an essential role in cell adherence to surfaces of biomaterials.^84^ Bordoni et al. used carboxymethylated negatively charged and enzymatically degraded noncharged nanofibrillated cellulose (NFC) to observe the influence of charged functional groups on cell adherence.^65^ More cells were found to be attached to noncharged NFC owing to repulsion between the biomaterial surface and cells.

The compatibility of bioink with the bioprinting methodology and the bioink characteristics for optimizing bioprinting conditions should also be taken into consideration. The bioinks with shear-thinning features are usually employed for extrusion-based techniques as these properties facilitate the extrusion of bioink from the printing nozzle.^14^ Shear-thinning, which occurs in non-Newtonian fluids, can be described as a drop of viscosity with increasing shear rate, and it is an important parameter for decreasing shear stress.^85^ Most of the above-mentioned biomaterials with a wide range of viscosities (30 to 60 × 10^7^ mPa·s) apply to extrusion-based bioprinting methodologies.^86,87^ However, the use of low viscous biomaterials with this method causes decreases in the scaffold integrity. This can be compensated by increasing the deposition speed of bioink, but enhanced shear stress decreases the viability of the cells.^67^ Shear stress has a huge impact on cell viability, SCs differentiation, and cell interactions.^14^ Therefore, printing conditions including pressure, temperature, bioink viscosity, and nozzle diameter should be optimized due to their effects on shear stress. Many studies reported that cell viability is generally at 40–80% as a result of extrusion-based bioprinting-induced shear stress.^69^ Still, over 85% viability rate was reported for GSCs and glioma cell line U118 in gelatin, alginate, and fibrinogen hydrogel scaffolds.^31,88^ Extrusion-based bioprinting processes have a distinct benefit in the use of bioinks with high cell density (>10^8^ cells·mL^–1^).^69^ For example, this technique produced a 3D HA-based model encapsulating human fetal primary astrocytes (FPAs) with good resolution using high cell density (2 × 10^6^ cells·mL^–1^).^89^

The properties of bioink used for droplet-based bioprinting are mechanical stability, biocompatibility, biodegradability, and low viscosity.^90^ The bioink should be rheopectic in which viscosity increases over time with shear stress, thus inducing droplet formation.^14^ These properties restrict the number of usable biomaterials for this method. Collagen, agarose, alginate, and GelMA have been utilized successfully with this methodology to produce GBM and NB models.^33,34,39^ A 3D NB model was created by the droplet-based bioprinting method using collagen which is profusely found in this tumor.^34,39^ The bioink made of 0.2% collagen and 0.5% agarose was found to be suitable for bioprinting while the bioink containing 0.3% collagen is not appropriate for bioprinting since this bioink may lead to clogs in the bioprinter head and its solid-like structure is related to shear stress which affects cell viability and rheology of the bioink.^39^ The necessity of using low viscous materials (3.5 and 12 mPa·s) due to clogging issues requires the use of cross-linking biomaterials in bioink to solidify the structure right away in a layer-by-layer manner.^67^ This necessity also limits the cell population that can be employed in the bioink. Thus, cell densities lower than 10^6^ cells·mL^–1^ were suggested with this methodology.^33^ Nonetheless, a recent study successfully fabricated the GBM model including GelMA with 20 × 10^6^ cells/mL cell density resulting in viability over 90%.^33^

Laser-based bioprinters can be used with bioinks with a wide viscosity range (1 to 300 mPa·s) and high cell densities (10^8^ cells·mL^–1^).^87,91^ Alginate, collagen, fibrin, and Matrigel have been utilized with this modality for tissue engineering applications.^92^ Since this technique is nozzle-free, cells do not experience mechanical stress, resulting in a high cell viability of 95% in scaffolds.^67^

SLA- and DLP-based processes are operated through the photopolymerization of light-sensitive hydrogels.^70^ Light-sensitive biomaterials, compatible with these methodologies, are glycidyl methacrylate hyaluronic acid (GMHA), GelMA, and poly(ethylene glycol) diacrylate (PEGDA).^55^ Another UV-curable hydrogel, silk fibroin (Sil-MA), was also found to be compatible with the DLP-assisted 3D bioprinting technique ensuring the development of complex models like the brain.^93^

Natural biomaterials like gelatin can be synthetically changed to achieve control over the biochemical and mechanical properties of the construct, such as degradation and gelation time.^15^ GelMA, which is formed with the interaction between gelatin and methacrylic anhydride (MAA), has drawn great attention lately due to its enzymatic degradability and biocompatibility.^70^ GelMA hydrogels also have similar features to neural tissues, such as permeability and water content. GelMA was utilized as a photosensitive material for the development of GBM models using the DLP approach.^36,49,94^ For example, a study that used the DLP strategy to create a GBM model using GelMA with a variable amount of HA reported high HUVEC and GBM cell (U87 MG) viability (more than 90%) even when cells are exposed to UV for a long time (1000 s).^94^

Low-viscosity hydrogels have been reported to be more compatible with SLA-based 3D-bioprinting methods.^70^ One significant drawback of SLA is the requirement of the bioink to be transparent with minimum scattering or light cannot pass the bioink efficiently leading to a nonuniform cross-linking. Therefore, the number of cells within the bioink is constrained to approximately 10^8^ cells·mL^–1^.^69^ A neural construct was produced using the SLA-based 3D-bioprinting method using nanobioink including GelMA and bioactive graphene nanoplatelets with pheochromocytoma (PC12) and NSCs. The viability of these cells in 3D bioprinted neural structure at low GelMA concentration was maintained for 2 weeks.^95^

Unlike SLA and DLP, CAL is capable of bioprinting bioinks by high viscosity (up to 9 × 10^4^ cP) and molecular weight.^70^ Similar to SLA and DLP, the compatibility of this strategy is constrained to light-sensitive biomaterials.^96^ This approach improves the viability of cells by refraining stress stemming from the layer-by-layer bioprinting approach.^70^

Several biomaterials such as laminin, collagen, and PEG-based hydrogels have been efficiently used with two-photon lithography.^55^ This approach was used to generate a free-standing poly(ethylene glycol) diacrylate (PEGDA) hydrogel scaffold that supports the growth of neuro2A cells.^97^ As a result, cells were shown to proliferate significantly and F-Actin microfilaments and expression of β-tubulin neuronal markers were reported. To our knowledge, no study on glioma and NB using this type of bioprinter has been published. This might stem from the fact that photoinitiators employed in two-photon bioprinting have been demonstrated to be taken up by cells leading to cytotoxicity with light exposure.^18^

Although many studies used the biomaterials above, the fact that ordinary 3D bioprinted models cannot demonstrate any response against external and internal stimuli unlike biological tissues limits the use of 3D bioprinting methodology. Recently, smart materials (e.g., shape memory polymers, composites, liquid crystal elastomers, multimaterials) have drawn great attention as they can alter their shape against chemical (e.g., ion content, pH), physical (e.g., temperature change), or biological signals.^98,99^ 4D bioprinting, where smart materials are combined with the 3D bioprinting technique, allows the more precise recapitulation of biological tissue dynamics by producing constructs that can change their characteristics according to surrounding stimuli.^98^

#### 2.3.2 Physical Properties of Hydrogel

In addition to biocompatibility and biodegradability, the key features of hydrogels to reflect malignant neural tissue are mechanical traits, porosity, and bioactive factors for promoting cell proliferation and function.^15^ Stiffness is a significant mechanical feature that has an impact on both neural cells and structure.^19^ The scaffold characteristics including concentration, cross-linking, density, and porosity influence stiffness which is demonstrated to influence cell migration, growth, cell signaling, and survival.^100,101^

Several studies prepared hydrogels whose elastic modulus is similar to that of neural tissue, i.e., in the range between 0.5 and 14 kPa.^102,103^ Proliferation rates of NB cell lines (SK-N-BE(2), SH-SY5Y) cocultured with Schwann cells change depending on the stiffness of materials containing methacrylated alginate (AlgMA) and GelMA.^50^ SH-SY5Y cells displayed an increased proliferation rate with decreased stiffness. SK-N-BE cells, on the other hand, demonstrated an enhanced proliferation rate in stiff bioinks. While Schwann cells decreased the proliferation rate of SK-N-BE(2) cells encapsulated in a 3D bioprinted NB model made of stiff bioinks, they did not alter the growth of these cells in the case of soft bioink. Moreover, Schwann cells encumbered the SH-SY5Y proliferation in both models, particularly in the soft model.^50^

3D bioprinted GBM constructs were developed employing HA derivatives encapsulating human patient-derived GBM SCs (TS576).^36^ While the genes related to pro-neural and mesenchymal subgroups upregulated in the stiff matrix, the soft matrix resulted in the enrichment of a gene related to the classical GBM subgroup. Additionally, the stiff structure supported aggressive mesenchymal GBM subgroup properties including stemness, angiogenic potential, and hypoxia.^36^ The SCs tend to turn into glial cells with an elastic modulus of more than 1 kPa while they are more likely to become a neuron with a softer matrix (100–500 Pa).^19^

Water uptake is another important feature of hydrogels. Although the high-water uptake capability of biomaterials mimics the hydrophilic behavior of ECM, excessive swelling may be damaging to the hydrogel structure.^104^

Porosity is a significant factor for neural ECM as it ensures effective nutrient supply and cell migration.^19^ GelMA was mixed with changing concentrations of AlgMA and SK-N-BE(2) cells to compare mechanical properties.^105^ The increase in alginate concentration led to the matrix being stiffer and thus less porous. Additionally, variations in pore size over time were observed. However, these changes were reported to be inversely proportional to stiffness, mRNA metabolism, cell density, and antiapoptotic behavior. As a result, GelMA without AlgMA and with 1% AlgMA were found to be the most desirable concentrations that reflect the structure of the high-risk NB types.^105^

### 2.4 Bioprinting Conditions

Bioinks with low viscosities allow cells to reorganize.^106^ In the extrusion-based methods, the bioinks with low viscosities are difficult to bioprint as this process entails low pressures and also may cause the development of unstable structures. Also, bioinks can overflow beyond specified limits throughout bioprinting. Viscous bioinks allow structures to be mechanically stronger and more resistant to possible deformities.^107^ However, highly viscous bioinks constrain cell survivability, decreasing their bioactivities in the scaffolds. Furthermore, higher extrusion pressure is needed for viscous bioinks to ensure a continuous flow rate, which leads to increased cell apoptosis. It is critical to tune the viscosity of hydrogels based on the bioprinting modality. Viscosity depends on molecular weight and concentration of biomaterials and temperature.^91^ Also, viscosity can be modified by developing composite hydrogels as demonstrated in some studies that investigated varying concentration levels of alginate–gelatin and collagen–alginate to identify the optimum range for viscosities.^108,109^ Moreover, changing viscosity based on concentration variation allows modification of stiffness.^110^

Temperature is an essential parameter for extrusion-based bioprinting since extreme temperature drops and rises might be detrimental to cells during bioprinting.^44^ The temperature ranges between 30 and 40 °C and 20 and 30 °C were selected by most studies that aimed to design extrusion-based 3D constructs containing cells due to the incubation time of cells (37 °C).^110^ The biomaterials including gelatin, alginate, GelMA, and agarose were employed by 65% of these works.^110^ The temperature in the heating source of thermal-inkjet bioprinters reaches 100–300 °C. Even though this caused concerns for cell viability in bioink, the latest studies demonstrated that high temperatures do not have a significant effect on cells as they are not subjected to high temperatures for a long time.^111^

Pressure is also a very important parameter that changes regarding bioink viscosity in extrusion-based processes. While low pressure (∼45–70 kPa) resulted in high cell viability (∼100%), higher pressure (>190 Pa) decreased cell viability to <65%.^67^ The impact of temperature, pressure, and concentration of gelatin and sodium alginate on the viability of NSCs, SH-SY5Y, and iPSCs was studied.^44^ The blend with 4% gelatin and 6% sodium alginate bioprinted at a pressure of 45–70 kPa and temperature of 25 °C was found to be optimum. While almost all iPSCs and NSCs remained viable for 7 days, ∼50% of SH-SY5Y cells in the 3D bioprinted construct with 6% sodium alginate and 4% gelatin were viable after 5 days. Another study, that employed cellulose nanofibrils and carbon nanotubes, developed a 3D model with an extrusion bioprinter at 65 kPa pressure.^83^ Moreover, a recent study employed 1 kPa pressure on HA and poly(ethylene glycol) based bioink containing SH-SY5Y cells and obtained over 85% cell viability 1 day after bioprinting.^52^

It is significant to note that the results of the bioprinting technique tend to vary with any slight change in parameters. Based on the type of bioprinting modalities, the parameters that have a direct effect on the characteristics of bioprinted models alter. For instance, while pressure might be the significant parameter for extrusion-based bioprinting, the effect of energy source might be significant for the droplet-based bioprinting approach. Despite the studies that attempted to standardize the bioprinting parameters, further investigations and databases addressing this problem in a broad scope, rather than focusing on only one particular tissue, process, bioink, or characteristic, like structure integrity or cell survival, are required.^75^

### 2.5 Cross-linking

Cross-linking is a crucial step in the fabrication of 3D bioprinted constructs as it affects the physicochemical and mechanical features of the 3D bioprinted structures and interactions of cells found in the hydrogel.^112^ The cross-linking method that is selected based on the functional groups and polymeric backbone of bioink materials can be realized through physical, chemical, and enzymatic procedures or by using them altogether.^112^ This process can be carried out before, during, and at the end of the bioprinting.^113^ The type and timing of cross-linking depend on the bioprinting methodology and biomaterial. As the droplet-based strategy requires the use of low-viscosity bioinks, rapid cross-linking procedures are needed to facilitate bioprinting.^91^ The procedures where bioink is cross-linked immediately after ejection (*in situ* cross-linking) are preferred to prevent blockages in the nozzle. On the other hand, cross-linking in the extrusion-based approach can be realized after bioprinting since the viscous bioinks preserve structure integrity after deposition.

#### 2.5.1 Physical Cross-linking

Bioinks that can be cross-linked physically are very appealing in the extrusion-based bioprinting method since they can be used with minimal effect on cell viability.^112^ However, most models obtained utilizing physical cross-linking are fragile. Hence, this approach is mostly employed for soft constructs that reflect tissues like the ones of the lung or brain. The well-known hydrogels that can be cross-linked physically include gelatin, collagen, Pluronic (F-127), Matrigel, chitosan, alginate, and agarose.^19^

One of the common processes in physical cross-linking is ionic interaction where multivalent cations are added to the hydrogel to stimulate gelation.^112^ Various multivalent cations such as calcium, zinc, barium, strontium, and ferric can be employed to cross-link to alginate-based hydrogels.^114,115^ Ca stands out as a cross-linker among others as it renders the biological characteristics of 3D models more stable.^116^ CaCl_2_ was found to be better at maintaining Schwann cell viability compared to BaCl_2_ and ZnCl_2_.^116^

Many studies on 3D bioprinted GBM^30,31,45,88^ and NB models^58,117^ that employed alginate-based hydrogels and an extrusion-based strategy utilized CaCl_2_ as a cross-linker which has high solubility and induces fast gelation.^112,114^ CaSO_4_ is reported to make 3D constructs considerably stiffer than other cross-linkers and lead to slow gelation of alginate.^112,114^ CaSO_4_ was employed to cross-link electrically conductive nanofibrillated cellulose, alginate, and carbon nanotube-based bioink including SH-SY5Y cells.^65^ Electrical conductivity, which was found to enhance neural differentiation, can be adjusted by cross-linking alginate through Ca.^65^

Ionic cross-linking can be also performed without metal ions by using the electrostatic interaction of ions that are found in polymer chains. In this method, two hydrogels with opposite charges are selected to create an electrostatic force.^118^ Hydrogels are divided into three types regarding ionic charges, which are cationic hydrogels such as chitosan and gelatin, anionic hydrogels like alginate, and neutral hydrogels such as sulfobetaine and dextran.^119,120^ Three cationic hydrogels, including GelMA, gelatin, and chitosan, and three anionic hydrogels, including xanthan, alginate, and K-carrageenan (Kca), were studied to understand the impact of differently charged materials on the extrusion-based bioprinting process.^121^ The hydrogel with GelMA (10 wt %) and Kca (2 wt %) came out as the most desirable blend in terms of maintaining effective electrostatic force.

#### 2.5.2 Chemical Cross-linking

Chemically cross-linked bioinks are mechanically more stable than physically cross-linked bioinks.^19^ Photo cross-linking is one of the common chemical cross-linking approaches. Poly(ethylene glycol) diacrylate (PEGDA) and GelMA are mostly preferred materials that can undergo photopolymerization.^19^ In light-based 3D bioprinting approaches, cross-linking is realized with free-radical polymerization of photocurable bioink.^55^ When bioink is exposed to light, absorption of energy by photoinitiators generates reactive species that induce a photopolymerization reaction, allowing the production of covalently cross-linked bioink.^70^ Photopolymerization is also utilized *in situ* or post-bioprinting via droplet and extrusion-based bioprinting techniques to cross-link the bioinks/scaffolds.^111^ Several studies that developed DLP/extrusion-based 3D GBM and NB models utilized GelMA and cross-linked their constructs with UV light.^48,49,57,94,105,122^ However, while UV light is a frequent photo-cross-linking technique, it can lead to DNA damage due to light radiation or toxicity related to photoinitiator.^113^

Thermal cross-linking where bioinks are subjected to cold or heat is another approach for cross-linking. For instance, to encapsulate murine NSCs, polyurethane-based hydrogels were thermally cross-linked.^123^ Methylcellulose, agarose, collagen, and HA are some other common thermally cross-linkable materials that have been employed in several bioprinted models.^112^

Chemical reactions like azide–alkyne cycloaddition also induces the formation of covalent bonds in polymers that can be activated chemically.^118^ Some works combined HA with Bicyclo[6.1.0]nonyne (BCN) reagent and then cross-linked with azide-functionalized PEG (PEG-Az8).^89,124^ This blend promoted the proliferation of SH-SY5Y,^124^ human FPA, and U87 GBM cells.^89^ However, PEG-Az8 reduces bioprinting time to a few minutes as it starts cross-linking right away once it is added to hydrogel.^125^ Furthermore, unlike the other cells that demonstrate no large-scale interaction, FBA cells were observed to interact more actively with other cells in this hydrogel.^89^

Several works also preferred genipin as it is a nontoxic natural cross-linker, induces differentiation of hiPSCs, and increases the stability of fibrin.^25,26,126^

#### 2.5.3 Enzymatic Cross-linking

Enzymatic cross-linking where enzymes are utilized to create covalent bonds between protein-based materials has recently become prominent owing to its simplicity and nontoxic properties, unlike chemical cross-linkers.^118,127^

Fibrinogen is cleaved by thrombin, resulting in the formation of fibrin which is a cross-linked type of hydrogel.^19,128^ Therefore, fibrinogen and thrombin were used altogether in the studies that created 3D bioprinted GBM and NB models.^6,23,26,129^ For instance, a bioink including fibrinogen, genipin, and alginate was cross-linked with a blend containing thrombin, chitosan, and calcium chloride.^26^ Then, N-cadherin antagonist was added onto extrusion-based 3D bioprinted GBM models encapsulating U87 GBM cells and astrocytes, leading to considerable cell death relative to controls.

Transglutaminase, which is found abundantly in nature, has been identified as a nontoxic cross-linker of protein-based materials.^130^ Transglutaminase can catalyze covalent bonds within gelatin.^22^ Various works employed transglutaminase to cross-link their hydrogel-containing gelatin in their 3D bioprinted GBM and NB models.^6,22,78,131^ A double cross-linking method where alginate and gelatin were cross-linked with Ca and transglutaminase, respectively, was investigated to enhance biochemical features like cytocompatibility of biomaterials and physiochemical traits including stiffness, water holding capacity, and structural integrity.^131^ The double cross-linked model was better than the blends treated with only one cross-linking agent in terms of structural integrity. This model also had great water-holding capacity and maintained the viability of SH-SY5Y cells for more than 2 weeks.^131^

## 3 Characterization of Cells in 3D Bioprinted Models

The robustness of 3D bioprinted models can be validated by determining the metabolism and viability of cells. In 3D models, more complex characterization methods are required compared to 2D models. Imaging techniques enable researchers to observe cell morphology, their interaction with other cells, and viability.^132^

Common imaging techniques used in glioma models are light microscopy, fluorescence microscopy, and electron microscopy techniques. Optical/light microscope can be employed to observe the morphology and size of cells by maintaining the construct and cell network.^50,105,113^ The optical microscope was utilized to evaluate porosity, clustering density, Karyorrehexis index (the number of cells going through karyorrhexis or those in mitosis), apoptotic activity, and proliferation of SK-N-BE(2) NB cells using Ki67 marker in paraffin-embedded hydrogels consisting of AlgMA and GelMA.^105^ There are various stains to observe the cells: hematoxylin (DNA) and eosin (proteins) to visualize dead and live sections, Masson’s trichrome (TM) for staining collagen constructs like fibrosis in blue, toluidine blue to illustrate sections with abundant DNA and RNA, and Trypan blue to highlight dead cells.^113^

Fluorescence microscopy is a better option for chromatic staining as it is challenging for thick materials. This technique is utilized to visualize subcellular compartments such as mitochondria, cytoskeleton, nuclei, and other components.^23,24,113^ Antibodies or markers with fluorescent probes are used to observe cellular states. These are KI67 for cell proliferation, p-casp3 as an indicator of cell arrest, p16 or β-galactosidase for cellular senescence, and HIF1-α, EF5, and pimonidazole for indicating cells in hypoxic areas.^113^ Calcein acetoxymethyl stain, ethidium homodimers, and propidium iodide are widely utilized to highlight viable and dead cells, respectively.^6,26,57,64^ Propidium iodide was used to determine viability, and stained cells were observed by a confocal laser scanning microscope in a 3D bioprinted GBM model.^24^ Confocal imaging is an enhanced fluorescence technique that can image toward the depth of the tissues with good resolution.^133^ These characteristics render this microscope desirable for visualizing 3D bioprinted models.^29,37,81,124^ The viability of SH-SY5Y cells in alginate and gelatin hydrogel was evaluated using a laser confocal microscope after acridine orange (AO)/propidium iodide staining.^131^

Another study utilized a confocal microscope to visualize morphological variations of SH-SY5Y cells during differentiation.^134^ The images obtained by a confocal microscope displayed that the cellulose-based scaffolds with carbon nanotubes and undergoing the carbonization process promoted cell growth and differentiation more than untreated cellulose material.

Electron microscopy techniques allow nanoscale scanning for the surface or inner parts of structures.^113^ Scanning electron microscopy (SEM) analysis allows for observing the cell migration, morphology, porosity, attachment, and interactions of cells with each other as well as with the scaffold.^132^ Numerous GBM^22,25,46,89^ and NB^129^ models were evaluated using SEM. Some studies^32,88^ employed a transmission electron microscope (TEM) in addition to SEM to evaluate the ultrastructural variations of GBM SCs (3D-GSC23 cells). 3D-GSC23 cells were demonstrated to create spheroid-like constructs in a hydrogel consisting of sodium alginate and gelatin. TEM analysis displayed that 3D-GSC23 cells have an expanded nucleus which is related to biological features of tumor cells.^32^ Another study harnessed a novel imaging modality, second-generation mesoscopic fluorescence molecular tomography to visualize their patient-derived GBM models before and after TMZ treatment.^37^ This advanced imaging method offered better imaging of thick models in a short time without photodamaging samples.

Fluorometric or colorimetric approaches can also be used to determine the proliferation, metabolism, and viability of cells in bioinks.^113^ Alamar Blue and Presto Blue have been employed by various studies constructing 3D bioprinted glioma^22,37^ and NB models.^52,57,89^ These nontoxic methods are also preferred for the determination of cell counts.^19,113^ Furthermore, CCK-8, MTT, MTS, WTS, and XTT tests are widely employed to assess and observe the survivability, growth, mitochondrial activity, and viability of cells.^113,132^ Viable cells reduce tetrazolium salt compounds and produce formazan which is detected by absorbance.^19^ As these tests are noxious for cells, these procedures should be carried out as a last step. The cytotoxic effect of hydrogels containing cellulose nanofibrils and carbon nanotubes on SH-SY5Y cells was tested using MTS.^134^ While both hydrogels containing cellulose and cellulose nanofibrils were determined to be nontoxic for cells by showing cell viability over 90%, their counterpart including carbon nanotubes decreased the viability to 40% which is lower than the viability threshold (70%) for the cytotoxicity test.^134^

Additionally, the CellTiter-Glo 3D kit specifically developed for 3D models has been employed for viability quantification of GBM and endothelial cells in HA-based hydrogels.^36^

Finally, flow cytometry provides an opportunity to perform several analyses including proliferation, viability, and quantification of anticancer compound uptake.^113^ The viability of IMR-32 NB cells in a chitosan-gelatin blend was determined by flow cytometry and a live/dead assay.^78^ Constructs demonstrated high viability after 5 days.

## 4 Applications of 3D Bioprinted Models of Brain (Intracranial) Tumors

In the past decade, many 3D bioprinted GBM models were developed to recapitulate TME and develop a drug screening platform where clinical outcomes can be predicted. Presenting a more realistic GBM microenvironment was the main objective in most studies to validate their approach that 3D models offer more realistic outcomes than 2D models. Some of these models were evaluated based on cell survival/proliferation, therapeutic response/resistance, and GBM-specific markers. The GBM microenvironment was also captured to observe the influence of macrophages on GBM progression and the crosstalk within cells and examine the relation of GSCs with cancer drug resistance and vascular formation by incorporating GSCs. Moreover, the link between GBM, endothelial cells, and vascular development was the focus of many studies to reflect the aggressive nature and progression of GBM. The use of different cells allowed for obtaining a better GBM environment as well as revealing the relation of them with GBM cells and ECM. Furthermore, the impact of ECM physical properties on the model was investigated to find the ideal conditions for cell survival, proliferation, cell differentiation, and structure integrity.

To the best of our knowledge, there are no studies that developed 3D bioprinted constructs for other brain tumors such as medulloblastoma (MB), ependymoma, and oligodendroglioma, while there are 3D models where spheroids were created to investigate these brain tumors.^135−139^ Brain tumor cells obtained from three medulloblastoma, one GBM, three ependymoma, and four astrocytoma patients were incorporated into a 3D silk fibroin-based scaffold.^139^ Genetic profiles of all spheroids and patient-matched tissue were determined and compared with each other. The transcriptomic signature of the 3D MB model in pro-neural and pro-endothelial cell growth media with scaffold was demonstrated to be the most similar to the patient tissue (less than 1% gene expression difference). This study is important as it brings us closer to personalized drug evaluation and shows that silk-based hydrogel supports tumor spheroids including astrocytoma which is known to be challenging to culture in vitro. However, this model is still devoid of essential materials of ECM components and various cell types including neurons, microglia, astrocytes, fibroblasts, endothelial cells, immune cells, and pericytes.^139^

**Figure 2:**
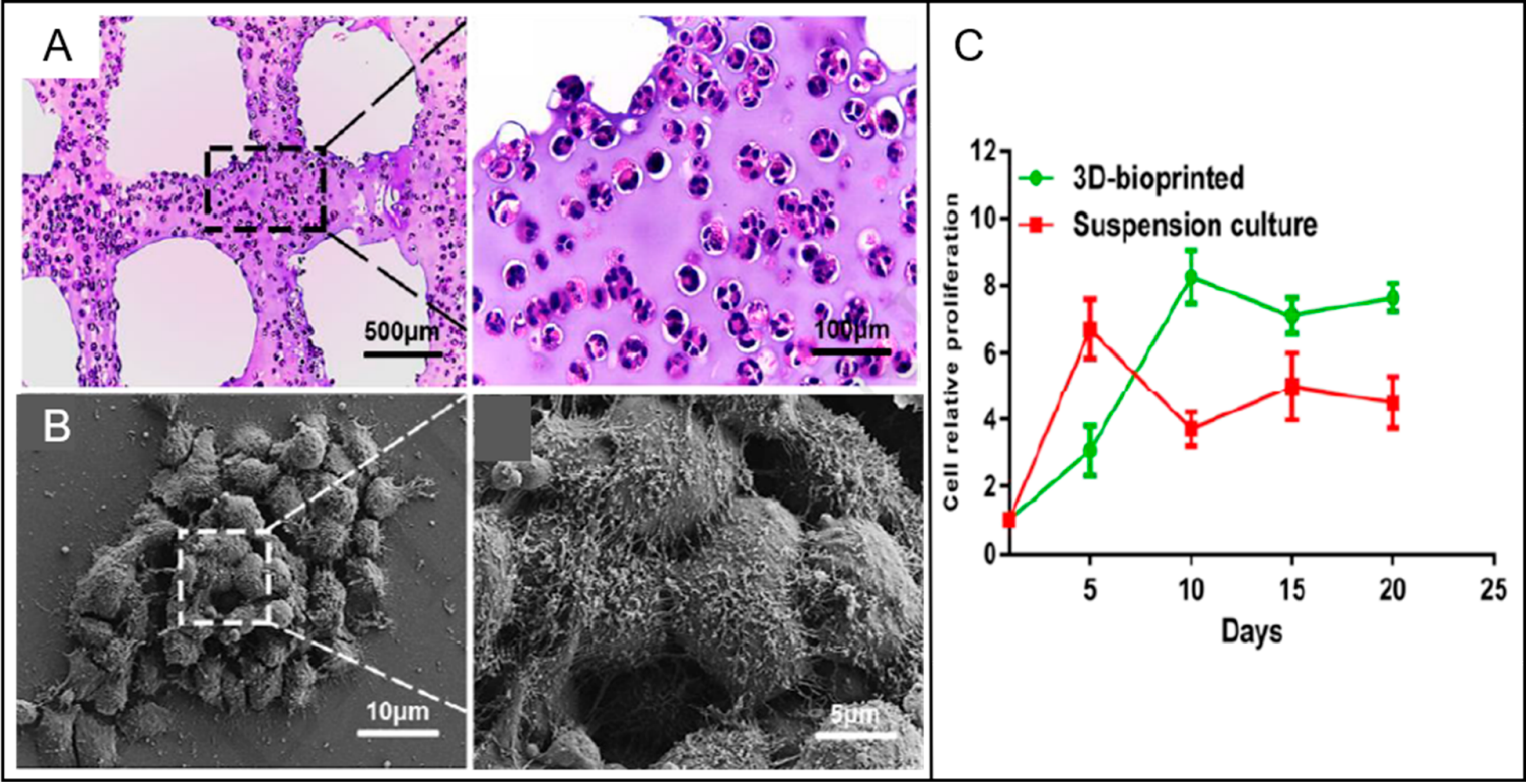
(A) Glioma stem cell spheroids in a 3D bioprinted grid scaffold stained with hematoxylin and eosin. (B) SEM images of glioma stem cell spheroids in 3D bioprinted scaffolds. (C) The proliferation rate of glioma stem cells in suspension culture and 3D bioprinted structures. Reproduced with permission from ref (88). Copyright 2018 Elsevier.

An early work fabricated a 3D bioprinted cancer model containing cancer cells and Matrigel employing a high-throughput cell patterning platform.^140^ Then, 3D bioprinted GBM models as grid patterns have garnered interest.^6,88^ 3D bioprinted grid scaffold composed of gelatin, alginate, and fibrinogen maintained the viability of GSCs with approximately 87% (Figure 2A,B).^88^ The proliferation rate of GSCs was also found to be more stable than the 2D culture of cells (Figure 2C).^88^ In another study, TME was recapitulated by developing grid scaffolds made of gelatin, alginate, and fibrinogen with GBM U87 and patient-derived GBM SCs.^6^ The potential of GBM SCs to form vascular tumors was confirmed with high expression of tumor angiogenesis biomarker, VEGF. The model was also found to be better in terms of reflecting the tumor environment due to its higher resistance to TMZ than 2D models.^6^

To generate scaffold-free neural tissue, the Kenzan technique was employed, where human neutrospheres containing iPSC-derived human progenitor cells were positioned in an array of needles and fused to develop a neural organoid.^29^ Subsequently, a spheroid including U118 human glioma cells was created using a bioprinter and placed on the neural organoid. After coculturing for several weeks and removing needles, GBM cell invasion through the neural microenvironment was observed efficiently using 3D confocal microscopy. This technique also allowed precise spatial control, showing an advantage of combining 3D bioprinting and neural tissue culture.^29^

**Figure 3:**
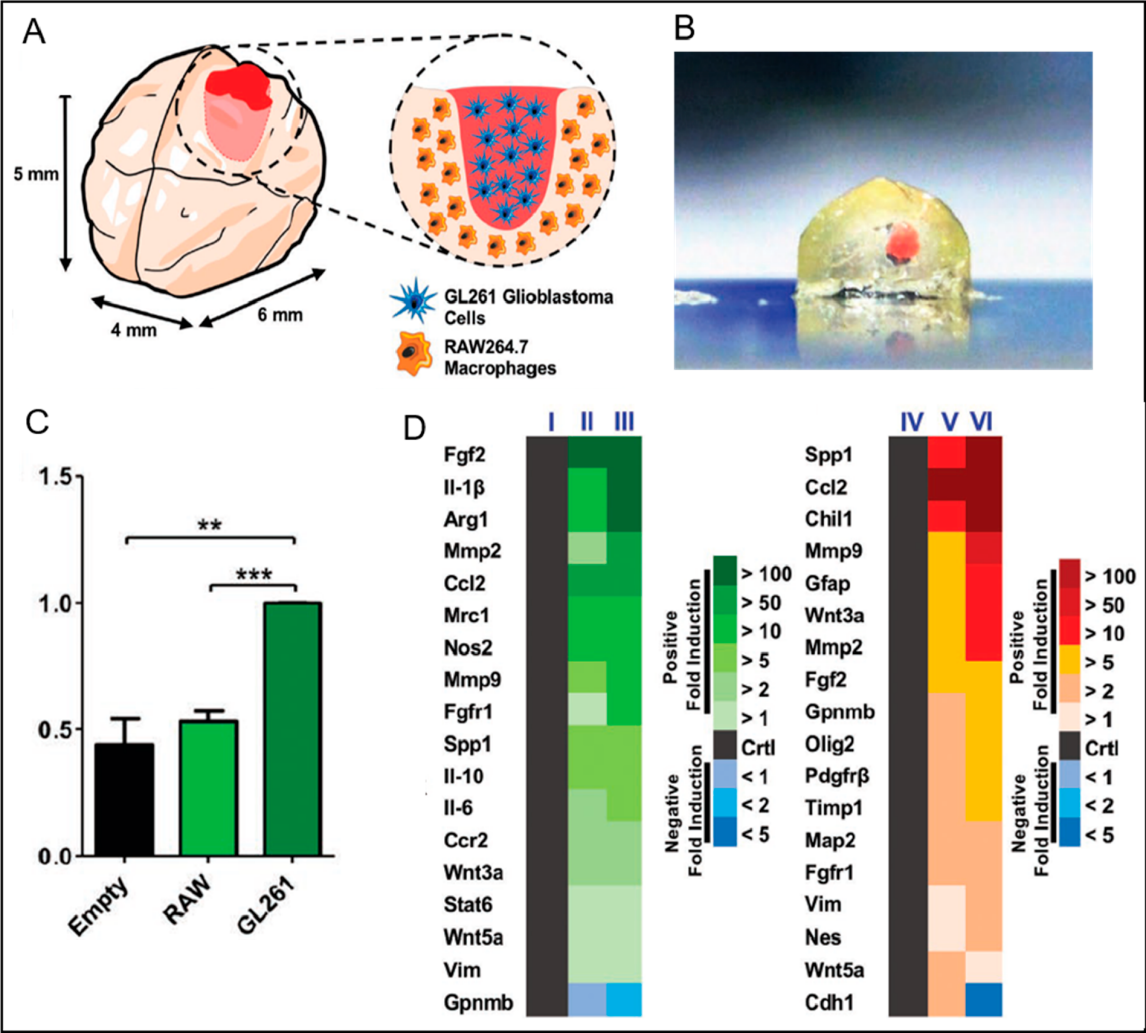
(A) Schematic representation of the mini-brain consisting of macrophages and GBM cells. (B) Bioprinted mini-brain (in 5 mm scale) containing the GBM region in red. (C) Demonstration of macrophage migration toward control, GL261 (GBM cells), and RAW264.7 (macrophages). (D) Illustration of gene expression in macrophages for (I) 2D model, (II) 3D bioprinted monocellular model, and (III) 3D bioprinted coculture model with macrophage/GBM cells and gene expression in GBM cells for (IV) 2D model, (V) 3D bioprinted monocellular model, and (VI) 3D bioprinted coculture model with macrophage/GBM cells. Reproduced with permission from ref (48). Copyright 2019 Wiley-VCH GmbH.

A mini-brain containing mouse macrophages was developed to elucidate the interplay between GBM and macrophages (Figure 3A).^48^ GBM cells were placed in a cavity found on this construct (Figure 3B). As a result, GBM cells recruited macrophages and rendered them GBM-associated macrophages (GAMs) by mimicking clinical situations (Figure 3C). Furthermore, as it was observed in in vivo models, GBM-specific markers were found to be highly upregulated in the 3D bioprinted model relative to its 2D counterpart (Figure 3D). It was also observed that cadherin expression reduced and vimentin and nestin expression increased, suggesting that GBM cells obtained characteristics of migration. The construct was employed to test chemotherapeutic and immunomodulatory compounds and displayed clinically relevant properties.^48^

**Figure 4:**
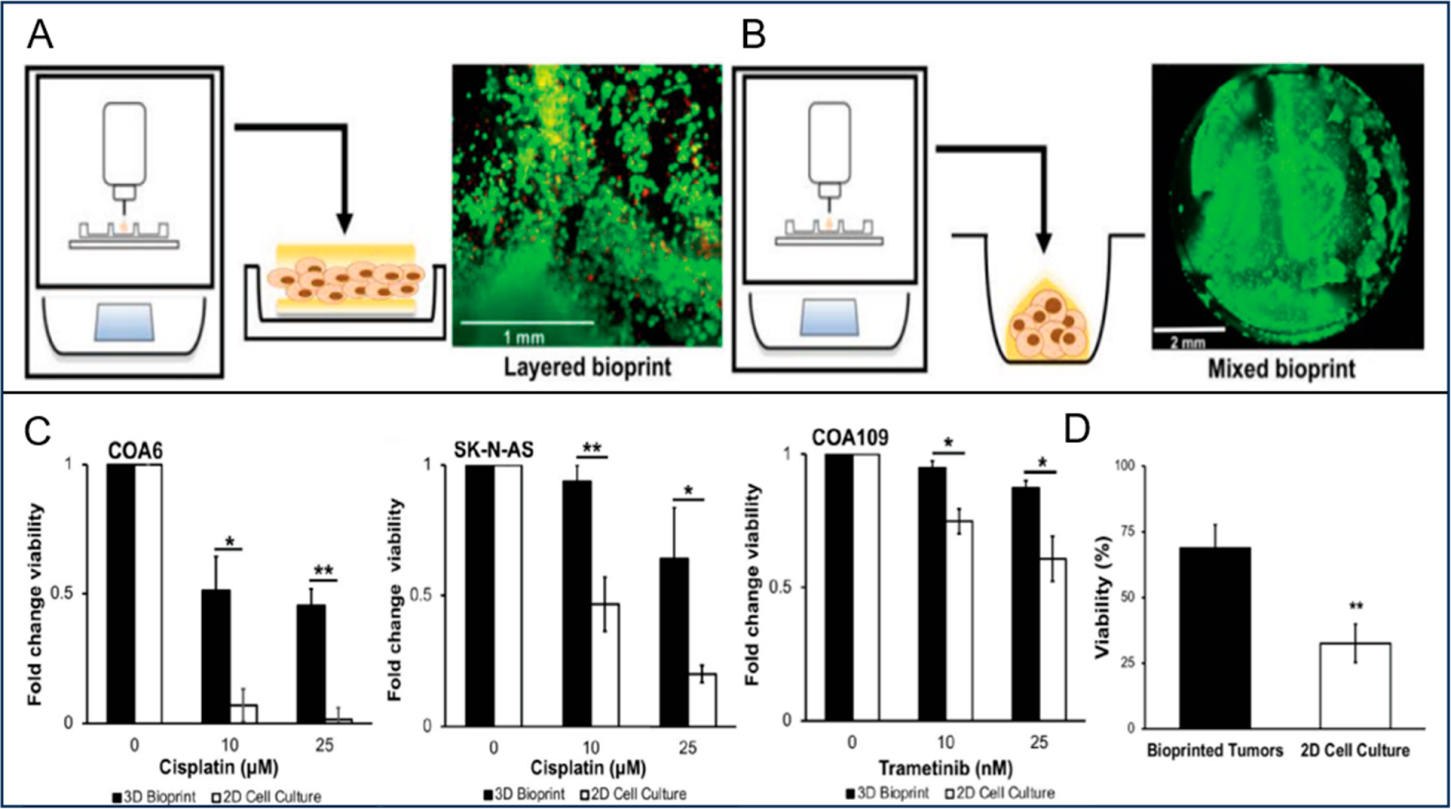
(A) Three-layered bioprinted model. Top and bottom layers consist of gelatin/sodium alginate hydrogel, and the middle layer has tumor cells. The quantification of viable and dead tumor cells in the layered model using Calcein AM (green) and SYTOX Orange (red), respectively. (B) Mixed bioprinted model that contains the blend of gelatin/sodium alginate hydrogel with tumor cells. The quantification of viable and dead tumor cells in a mixed bioprinted model using Calcein AM (green) and SYTOX Orange (red), respectively. (C) The influence of hypoxic conditions (under 1% oxygen) on the viability of SK-N-AS cells laden in 3D layered model and 2D culture. (D) The chemoresistance of patient derived xenograft (PDX) COA6, SK-N-AS, and human neuroendocrine PDX COA109 cells laden in 3D tumors (bioprinted on half of the 96-well plate) and in 2D cultures (placed in the other half of the 96-well plate). Reproduced with permission from ref (117). Open Access.

A very recent study constructed 3D bioprinted neural crest-derived solid tumors made of gelatin and sodium alginate with NB cell lines (SK-N-BE(2) and SK-N-AS), NB (COA3 and COA6), and high-grade neuroendocrine-like tumor (COA109) PDXs cells.^117^ Two models were created in this study. In the first model, a three-layer model where the top and bottom consisting of hydrogel and the middle layer composed of cells was fabricated (Figure 4A). In the second model, cells and hydrogels were homogeneously mixed (Figure 4B). Both layered and mixed bioprinted models maintained the viability of cells. Mixed bioprinted models were also injected into mice, and they were observed to grow after injection. Furthermore, 3D bioprinted models were found to be more resistant to chemotherapeutic drugs and hypoxia than 2D cell culture rendering 3D bioprinted models more desirable for preclinical trials (Figure 4C,D).^117^ While this model is a significant advance for this scope in terms of its superiority to 2D culture and its ability to mimic histology and immunostaining traits of original tumors and continue to grow in mice, the model did not contain noncancerous cells like HUVECS, fibroblasts, or cancer SCs that are highly associated with chemoresistance and disease reoccurrence.^117,141^

The 3D bioprinting/printing approach can also be used for other preclinical applications including drug delivery, toxicity efficacy, and assessment of pharmacokinetic parameters.^142^ In vitro 3D models have uncovered more biomimetic toxicities in pharmaceuticals than conventional 2D models.^142^ Therefore, 3D bioprinted models with highly biomimetic features can be counted as better alternatives for in vitro toxicity assessments than 2D or other 3D constructs. Biodegradable materials have enabled the fabrication of drug delivery systems with patient-specific doses and devices compatible with patients’ anatomical characteristics.^143^ Hydrogel meshes laden all-trans retinoic acid (ATRA)-loaded polymeric microspheres were produced using 3D extrusion-based printing.^144^

The strategy facilitated the ATRA release, which is controlled with material concentration and mesh porosity, thus resulting in enhanced drug uptake, and the apoptosis of GBM cells. This strategy was based on the brain implantation of the mesh that prevents displacement of microspheres by the cerebrospinal fluid.^144^

Another study developed a blood–brain barrier (BBB) model including mouse brain endothelial cell lines and microarrays constructed from collagen type I in an extrusion-printed frame that recapitulated BBB characteristics.^145^ The expression of tight junction protein ZO-1 enhanced in 2 weeks, and the transendothelial permeability was validated. The residence time of this system mimicked the real blood residence time in the brain, allowing prediction of the drug permeability for clinical trials if combined with a GBM model.^145^

### 4.1 3D Bioprinted Vascular Models

3D bioprinting techniques possess the capability to manage different cell/tissue types with spatially controlled deposition, thus offering the development of intricate tissue geometries.^146^ Bioprinting a vasculature network is necessary for artificial tissue as it enables a pathway for delivering nutrients and removing wastes.^147^ Vascular tissues have several vessel systems with different morphologies and sizes changing from micrometer to millimeter.^148^ While capillaries possess a layer of endothelial cells (ECs), large vessels comprise three layers. The inner surface of vessels is lined by endothelial cells. The middle layer contains collagen fibers, elastic tissue, and smooth muscle cells (SMCs). The outer surface of the vessels is made of collagen fibers and elastic tissue.^149^ The type of ECs also varies according to the size of the vessels. HUVECs, induced pluripotent SC-derived endothelial cells (iPSC-ECs), and human microvascular endothelial cells (HMVECs) are the most widely employed EC types in tissue engineering approaches.^149^ HUVECs are the most commonly utilized endothelial cell type for 3D bioprinting vascularized models.^148^ In the context of vessel development, angiogenesis and vasculogenesis are the two most commonly investigated processes. Angiogenesis sustains malignant tissues by supplying nutrients and oxygen and taking wastes away.^150^ Vasculogenesis is carried out by recruiting endothelial progenitor cells (EPCs) that can transform into endothelial cells and invade the tumor to be directly involved in the development of tumor blood vessels.^151^

**Figure 5:**
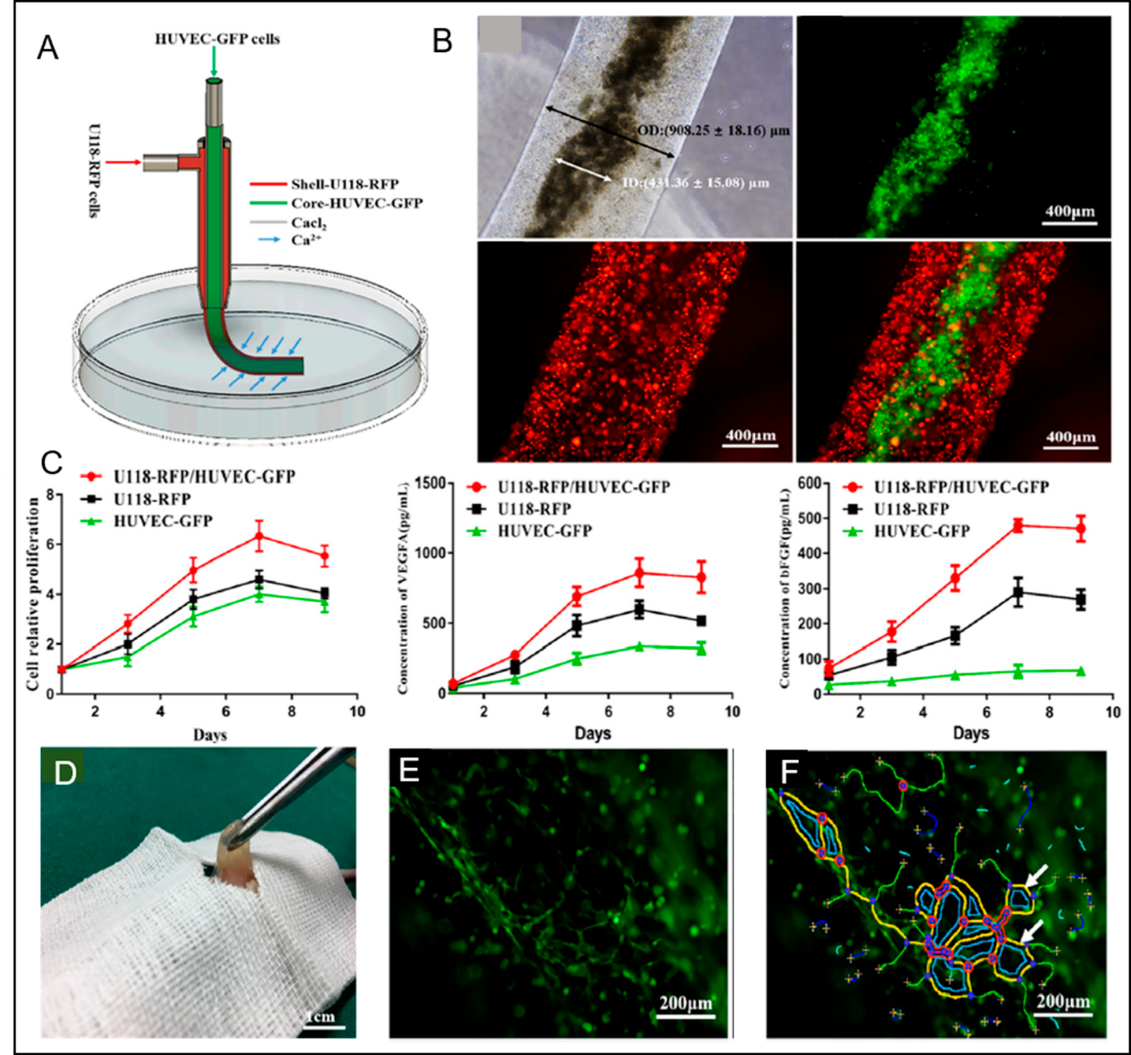
(A) Coaxial bioprinting setup of shell–core hydrogel microfibers with GBM and HUVECS cells. (B) Hydrogel microfibers consisting of shell and core that have GBM U118 cells (shown in red) and HUVECs (shown in green), respectively. (C) The comparison of proliferation rate and secretability of VEGFA and bFGF for HUVEC and GBM U118 cells alone and together. (D) The change in color and soft structure in the tumor shown are related to GBM morphology. (E, F) Illustration of tubular structures formed in the microfiber. Reproduced with permission from ref (152). Open Access.

A recent study elucidated the influences of bioprinted GBM cells on the vascularization potential of endothelial cells and their involvement in angiogenesis.^152^ Coaxial extrusion bioprinting was employed to fabricate core–shell hydrogel microfibers whose inner core consists of HUVECSs in collagen and outer layer includes human GBM (U118) cells in sodium alginate (Figure 5A,B). Using these cells together caused a higher proliferation rate than culturing these cells individually. Furthermore, the development of HUVEC tubule-like structures was determined to be more in the coculture of these cells than in the culture of HUVECs. U118 cells supported the vascularization of HUVECs by excreting vascular growth factors (Figure 5C). Moreover, hydrogel microfibers including U118 cells were transplanted into mice. The characteristics linked to human glioma morphology such as fish-like color change and soft structure were observed in xenograft tumors (Figure 5D). Additionally, vascular structure and origin of xenograft tumors were analyzed utilizing antihuman/mouse CD105 and human-specific antiCD105. It was found that 22% and 78% of the tumor CD105+ cells were human and mouse origin, respectively, suggesting that cancer cells can recruit host vascular endothelial cells to be involved in tumor angiogenesis as well as directly taking part in the angiogenesis themselves. Tubule-like forms containing endothelial/glial phenotypic cells were also detected in xenograft tumors indicating that U118 cells could transdifferentiate or fuse with endothelial cells to play a role in tumor angiogenesis (Figure 5E,F).^152^

Glioma and GSCs promote glioma angiogenesis by converting into endothelial cells or excreting VEGF.^32^ A study examined the role of GSCs in tumor vascularization on the GSC tumor model which was developed using a 3D multinozzle bioprinter.^88^ A bioink including gelatin, alginate, fibrinogen, and GSCs was employed. GSCs were found to be involved in tumor vascularization by excreting VEGF. Cell proliferation and characteristics associated with vascularization such as stemness were observed to be increased in the 3D bioprinted GSC model compared to the GSC culture.^88^

A very recent work employed several agents to examine the effects of pro- and antiangiogenic factors on microvessel networks.^129^ The bioink including fibrin, factor XIII, 5% GelMA, VEGF165, bFGF, and EGF, with patient-originated endothelial cells, NB cells, induced pluripotent SC-derived, and adipocyte-derived mesenchymal SCs was found to be most desirable in terms of vessel development and the bioprinting process.^129^

Bioprinted vascularized GBM models were also employed as drug screening platforms.^22,23,37^ A 3D bioprinted GBM model including perfused vascular channels for drug screening was developed by bioprinting a collagen layer between channels made of gelatin.^37^ HUVECs were cultured on channels to grow a cell lining on the surface of the inner channel. After TMZ application for 21 days, the suspended 3D patient-derived GBM spheroids demonstrated a higher reduction in metabolic activity level compared to the 2D monolayer model and also a decrease in tumor growth. Overall, this customizable system allows for testing therapeutic alternatives under improved physiological settings to determine treatment efficiency.^37^

Bioprinting vascular models particularly capillaries are mostly restricted owing to the resolution and speed of current bioprinters.^153^ The diameter of capillaries in the brain varies from 7 to 10 μm. The maximum resolution of extrusion-based and droplet-based bioprinters is ∼50 μm owing to the size of the nozzle and inkjet head.^9,154^ However, such structures with good resolution are generally obtained with biomaterials without cells. Using higher cell density in bioink entails a bigger nozzle, or it would be very challenging to preserve cell viability because of shear stress during bioprinting. In such cases, resolution might vary between 200 and 500 μm. The resolution in 3D light-based bioprinters ranges from a few tens to hundreds of micrometers considering the limitations such as light scattering caused by cells. Therefore, achieving high resolution (≤50 m) with high cell density (≥20 million cell/ml) in the development of complex models is an arduous task.^154^ A very recent study showed that it is possible to create vascular channel diameters between 250 and 600 μm with high cell density (40 million cells/mL) using DLP-based 3D bioprinting and iodixanol that decreases light scattering caused by cells.^154^ However, the fabrication of capillaries using the bioprinting approach is still an issue due to long-time requirements which have a direct impact on cell viability. Despite attempts such as the inclusion of angiogenic growth factors into bioinks or developing vascular structures using synthetic materials, further studies are required for vessels with a diameter of less than 5 mm.^153^

### 4.2 Tumor-on-Chips Integrated with a 3D Bioprinting Approach

Microfluidics employs small devices that allow the manipulation of fluids on a microscale via channels.^28^ Microfluidic “brain-on-a-chip” or “tumor-on-a-chip” platforms have been employed and demonstrated to be advantageous in pharmacological research, drug delivery systems, and toxicity tests.^9^ One of the most important benefits of this technology is the feasibility of modeling 3D complex vessel systems on hydrogel chips.^9^ These devices can also keep cells viable for a prolonged period of time by flowing culture through the parenchymal or the endothelium-included vascular channels alone or both.^155^ Fluid flow in contact with tissue influences cell arrest in cancer cells, and invasion of tumor cells in the direction of flow regulates proliferation and gene expression.^156^ Moreover, shear stress produced by flowing liquid in the microchannels mimics the stress created by the blood in the vasculature.^100^ Combining the benefits of microfluidic chips including perfusion and gas permeability with the 3D bioprinting approach allows the bioprinting of perfused and accurately positioned cultures with the desired structure for physiological research and drug screening at the organ scale.^157^

In the past decade, intricate and specific patterns like bioprinted GBM-on-a-chip models have drawn interest.^27,34,122,158^ A novel technique that combines microfluidic and inkjet bioprinting technologies was employed to fabricate a model including HepG2 liver and U251 GBM cells suspended in sodium alginate.^34^ The bioink was copatterned into channels of the chip using an inkjet bioprinter. Tegafur, which is a prodrug of 5-fluoro uracil with anticancer traits, decreased the viability of GBM cells. However, it was also reported that Tegafur suppressed the proliferation of malignant cells only in the presence of HepG2 cells. This model allowed spatially controlled deposition of cells into microchips. This method can be beneficial to improve the cell patterning efficacy in microfluidic chips and decrease the burdens of experimental studies.^34^

Oxygen gradients can also be generated in microfluidic chips recapitulating the impact of oxygen on tumor formation and metastasis.^159^ A GBM-on-a-chip model that maintains an oxygen gradient was constructed with ECM bioink obtained from the porcine brain.^27^ The ring consisting of three regions, core, intermediate, and peripheral, was filled with patient-derived GBM cell-laden hydrogel, HUVEC cell-laden hydrogel, and silicone, respectively. The whole structure except peripheral regions containing gas-permeable silicone was covered with nonpermeable glass to generate a radial oxygen gradient where oxygen molecules can only access the tumor core by penetrating silicone and HUVEC sections. While hypoxic cells enhanced from outer to core regions, proliferating cells behaved inversely. Invasive cells were displayed in the outer region owing to their tendency to migrate sections with more oxygen. To compare, cells were also cultured in collagen gel. Although both blends exhibited viability of more than 90%, invasion and proliferation activities of GBM cells in ECM derived from pig brains were higher. Also, angiogenesis factors expressed by HUVEC cells were determined to be more in this hydrogel relative to ones in collagen. The model demonstrated patient-specific resistance to TMZ and chemoradiation. This model offers a platform where effective treatments can be identified for cancer patients who develop resistance to traditional therapies.^27^

Another GBM-on-a-chip model was presented integrating microfluidic and 3D bioprinting approaches to evaluate the effect of microgravity on the tumor.^122^ The vascularized construct was obtained by depositing GelMA-alginate blend encapsulating GBM cells A-172 and GelMA-fibrin blend containing HUVECs in the core of the chip and ring shape surrounding the core, respectively. As a result, the lack of gravitational fields led to the inhibition of aggregation and migration of GBM cells. Even though this study did not test drug effects or intercellular communication, it demonstrated the potential of this platform in enabling biologically related properties that might enhance medical relevance, especially when it is integrated with spatially controlled deposition provided by 3D bioprinting technologies.^122^

## 5 Conclusions, Challenges, and Future Aspects

Brain tumors are one of the deadliest diseases that influence both adults and children. The most dangerous type of them is GBM with poor prognosis and low life expectancy under current treatments. The location, invasiveness, heterogeneity, and quick proliferation of GBM make the cure difficult. Furthermore, many patients become resistant to chemotherapy drugs after a while, mostly due to GSCs. Therefore, a platform where we can expand our knowledge about the characteristics and progression of GBM and try potential drug candidates is crucial. While 2D cultures have provided a myriad of information about gliomas in the past decades, they do not correctly mirror the tumor environment as well as the cellular crosstalk. Animal models also fail to reflect the biological and philological systems of humans correctly. Furthermore, using them raises ethical questions and it is very costly. 3D in vitro models have been introduced as a middle ground to overcome the shortcomings of the two systems. 3D bioprinting technology allows simultaneous deposition of more than one material, i.e., cell, ECM, and other biological components, and the development of intricate 3D structures. These 3D bioprinted systems can mimic the heterogeneity of tumors. Additionally, more complex structures with vascular development can be manufactured to supply oxygen and nutrients to cells and recapitulate angiogenesis or vasculogenesis. Combining bioprinting technology with microchips not only serves this type of objective but also allows us to control various parameters linked to gas and chemical concentration gradient, cell distribution, shear stress, spatial pattern of cells, and tissue–tissue interaction.

Despite the indisputable advantages of bioprinting technology, some limitations need to be addressed. The decrease in cell viability during bioprinting is a frequently encountered problem. Thus, flow rate, printing time, concentration of hydrogel, and pressure should be optimized to diminish cell damage. Preserving the stability of the construct is another challenging process during bioprinting. This procedure becomes much harder with the use of soft materials. To address this problem, the embedded 3D bioprinting and sacrificial framework strategies have been suggested to support the structure. It should be noted that the large number of variables in this system makes it difficult to find the optimum point for maintaining both the structure integrity and cell viability, resulting in loss of time and material. Furthermore, in artificial environments like in vitro systems, replicating the dense tumor stroma and ensuring identical cell subpopulations as observed in real (in vivo) systems remains a challenge. It is premature to unequivocally consider in vitro models as viable substitutes for in vivo models. Although verifying whether a model emulates in vivo pathological and physiological functions is crucial for translating any model, the complex structure of the system being constructed renders this verification very difficult in CNS models. To date, only a very limited number of studies have integrated both in vitro and in vivo models within a single developmental framework. Nevertheless, 3D in vitro models demonstrate the ability to forecast the effectiveness and performance of new therapeutics in an in vivo setting. While 3D in vitro models do not replace animal studies, they do diminish the need for such studies, making subsequent research more efficient and cost-effective. This shift in resources toward clinical phases can potentially accelerate the pace of clinical studies by optimizing time and budget allocation.

In this Review, common materials, cells used in this particular topic, the factors that have a significant impact on the fidelity of 3D bioprinted glioma models, cell interaction, viability, neural or SC differentiation, vascular formation, and GBM-specific properties were elaborated. Discussing 3D bioprinted models where brain cancer cell lines are harnessed was the other main point of this study. Additionally, 3D bioprinted models containing NB cell lines were covered as NB cell lines have the potential to differentiate into neurons; thus, they can be utilized in both 3D neural tissue and brain tumor models.

It was detected that most studies used commercial U-87 MG, U118-MG, and U251-MG GBM cell lines to manufacture 3D bioprinted GBM models. Harnessing patient-derived cells more than commercial cell lines will allow us to take one step closer to the field of personalized medicine and make significant clinical decisions in the future. Further investigation of GSCs by developing more individualized GBM models might be a good approach, as chemotherapy drug resistance is a serious problem. Moreover, the use of different cell types such as immune cells, astrocytes, endothelial cells, or vascular components will also contribute to better replication of heterogenic TME, as employing more than one cell and biological components influence protein excretion, growth, and drug response of cells. Overall, more sophisticated 3D bioprinted GBM models will expand our perspective on the progress and characteristics of GBM, limit animal trials, enable a realistic platform for drug screening, and bring us closer to personal therapy.

## Supporting Information Available

Tables S1-S1 and S2-S2 (the expanded versions of Table 1 and Table 2, respectively) containing key parameters of all studies (in the context of this review) that constructed 3D bioprinted GBM and neural/NB models using NB cells, respectively (PDF)
