# Supplementary material for: Applications of 3D Bioprinting Technology to Brain Cells and Brain Tumor Models: Special Emphasis to Glioblastoma
Source: ACS Biomater Sci Eng. 2024 Apr 26;10(5):2616–35. doi: 10.1021/acsbiomaterials.3c01569 (PMC11094688; doi:10.1021/acsbiomaterials.3c01569)
Supplement: Supplementary file 1 — ab3c01569_si_001.pdf [file ab3c01569_si_001.pdf]

Applications of 3D Bioprinting Technology to Brain Cells and Brain Tumor Models: Special Emphasis to Glioblastoma

Ilkay Irem Ozbek[1], Hale Saybasili[2], Kutlu O. Ulgen[1*]

Number of Pages: 17

Number of Tables: 2

Number of Figures: 0

Table S1-S1. Bioprinting Parameters for Glioblastoma Tumor Models.

Application	Cell type	Bioprinting technique	Bioink type	Crosslink Method	Viability Technique	Reference
3D bioprinted mini brains with Glioblastoma	Mouse Glioblastoma cells (GL261), Mouse macrophages cells (RAW264.7)	Custom-modified bioprinter	Gelatin methacryloyl, Gelatin	Photo-Induced Cross-Linking	Live/dead viability assay kit	1
3D bioprinted Glioblastoma vascular niche modelling	Human primary umbilical vein endothelial cells, Patient-derived Glioma stem cells		Collagen type I, Laminin			2
3D bioprinted Glioblastoma vascular modelling	Human primary umbilical vein endothelial cells, Glioblastoma cells (U87 MG), Lung fibroblasts	Extrusion bioprinter	Gelatine, Alginate, Fibrinogen	$CaCl_2$, Thrombin	Live/dead viability assay kit, Confocal microscopy	3
3D bioprinted Glioblastoma modelling	iPSC-derived human neural progenitor cells, Glioblastoma cells (U118)	Scaffold free bioprinter			Confocal microscopy	4
3D bioprinted Glioblastoma modelling	Glioblastoma cells (U87MG)	Extrusion bioprinter	Fibrin, Alginate, Genipin	$CaCl_2$, Chitosan, Thrombin	Live/dead assay kit, Fluorescence microscope	5

Table S1-S1. Bioprinting Parameters for Glioblastoma Tumor Models (Cont.).

Application	Cell type	Bioprinting technique	Bioink type	Crosslink Method	Viability Technique	Reference
3D bioprinted Glioblastoma vascular modelling	Human primary umbilical vein endothelial cells, Patient-derived Glioblastoma cells		Collagen, Gelatin		Alamar blue viability assay, Second-generation mesoscopic fluorescence molecular tomography, Wide-field fluorescence microscopy, Laser scanning confocal microscopy, Microscopic magnetic resonance imaging	6
3D bioprinted Glioblastoma modelling	Glioma stem cell lines (G144, G166 and G7), Glioblastoma cells (U87MG), Microglia, Monocytic cells	Multi-nozzle extrusion bioprinter	Alginate modified with RGDS cell adhesion peptides Hyaluronic acid, Collagen-1	$CaCl_2$	Live/dead viability assay kit, Confocal Microscope	7

Table S1-S1. Bioprinting Parameters for Glioblastoma Tumor Models (Cont.).

3D bioprinted Glioblastoma modelling	Glioma stem cell line (SU3), Glioblastoma cells (U87)	Multi nozzle extrusion bioprinter	Sodium alginate, Gelatin, Fibrinogen	Transglutaminase, $CaCl_2$ Thrombin	Live/dead viability assay kit, Fluorescence microscope	8
3D bioprinted Glioblastoma fiber modelling	Glioma stem cells (GSC23), Mesenchymal stem cells	Coaxial extrusion bioprinter	Alginate, Gelatin, Fibrinogen	$CaCl_2$, Thrombin	Live/dead viability assay kit, Fluorescence microscope	9
3D bioprinted Glioblastoma shell-core hydrogel microfiber modeling	Glioblastoma cells (U118), Glioma stem cells (GSC23)	Coaxial extrusion bioprinter	Sodium alginate	$CaCl_2$	Live/dead viability assay kit, Fluorescence microscope	10
3D bioprinted Glioblastoma modelling	Glioma stem cells (GSC23)	Multi nozzle extrusion bioprinter	Alginate, Gelatin, Fibrinogen,	$CaCl_2$, Thrombin, Transglutaminase	Live/dead assay kit, Fluorescence microscope	11
3D bioprinted Glioblastoma modelling	Glioblastoma cells (U118)	Multi nozzle extrusion bioprinter	Sodium alginate, Gelatin, Fibrinogen	$CaCl_{2,}$ Thrombin	Live/dead assay kit, Fluorescence microscope	12
3D bioprinted Glioblastoma modelling	Glioblastoma cells (U87MG), Human astrocytes	Extrusion bioprinter	Fibrinogen, Alginate, Genipin	CaCl2, Chitosan, Thrombin	Live/dead assay kit, Fluorescence microscope	13

Table S1-S1. Bioprinting Parameters for Glioblastoma Tumor Models (Cont.).

Application	Cell type	Bioprinting technique	Bioink type	Crosslink Method	Viability Technique	Reference
3D organ on chips Glioblastoma modeling	Glioblastoma cells (U-87 MG), Human primary umbilical vein endothelial cells	Extrusion bioprinter	Porcine Brain derived Extracellular matrix		Live/dead assay kit, Fluorescence microscope	14
3D bioprinted Glioblastoma modelling	Patient derived Glioblastoma cells, Human primary umbilical vein endothelial cells	Digital light processing based bioprinting	Gelatin methacryloyl, Glycidyl methacrylate hyaluronic acid	Photo-Induced Cross-Linking	Cell Titer Glo assay	15
3D bioprinted Glioblastoma modelling	Patient derived Glioma stem cells, Macrophages, Astrocytes, Neural stem cells	Digital light processing based bioprinting	Gelatin methacryloyl, Glycidyl methacrylate hyaluronic acid	Photo-Induced Cross-Linking	Cell Titer Glo assay	16
3D organ on chips Glioblastoma modeling	Hepatoma HepG2, Glioma cells (U251)	Droplet based bioprinter	Alginate	$CaCl_2$	Live/dead assay kit, Confocal microscope	17
3D bioprinted Glioblastoma modelling	Glioblastoma cells (U251 MG), Human primary umbilical vein endothelial cells	Droplet based bioprinter	Gelatin methacryloyl	Photo-Induced Cross-Linking	Live/dead assay kit, Flow cytometry	18

Table S1-S1. Bioprinting Parameters for Glioblastoma Tumor Models (Cont.).

Application	Cell type	Bioprinting technique	Bioink type	Crosslink Method	Viability Technique	Reference
3D bioprinted Glioblastoma vascular modelling	Human Glioblastoma cells (U87MG, T98G, and U373), Human embryonic kidney 293T cells, Human osteosarcoma (Saos-2) cells, MDAMB-231 Human breast cancer cells, GL261 murine GB cell line, microglia, Human primary umbilical vein endothelial cells, Murine pericytes, and astrocytes	Extrusion bioprinter	Fibrinogen, Collagen, Gelatin, Pluronic F127	Transglutaminase	Presto Blue Cell Viability Reagent	19
3D bioprinted Glioblastoma vascular modelling	Glioblastoma cells (U118), Human primary umbilical vein endothelial cells	Coaxial extrusion bioprinter	Collagen, Sodium alginate	$CaCl_2$	Alamar blue viability assay	20
3D bioprinted Glioblastoma vascular modelling	Glioblastoma cells (U118), Glioma stem cells (GSC23)	Extrusion bioprinter	Sodium alginate, Gelatin	$CaCl_2$	Live/dead assay kit, Fluorescence microscope	21

Table S1-S1. Bioprinting Parameters for Glioblastoma Tumor Models (Cont.).

Application	Cell type	Bioprinting technique	Bioink type	Crosslink Method	Viability Technique	Reference
3D bioprinted Glioblastoma organoid modelling	Human liver cancer cell line, human colorectal cancer epithelial cell line, Patient-derived Glioblastoma cells	Extrusion bioprinter (Immersion bioprinting method)	Methacrylated collagen type I, Hyaluronic acid, HyStem−HP hydrogel	Photo-Induced Cross-Linking	Live/dead assay kit and Macro-confocal microscopy	22
3D organ on chips Glioblastoma modeling	Glioblastoma cells (U-87 MG), Human primary umbilical vein endothelial cells	Digital light processing based bioprinting	Gelatin methacryloyl, Hyaluronic acid	Photo-Induced Cross-Linking	Live/dead assay kit and Confocal microscopy	23
3D organ on chips Glioblastoma modeling	Glioblastoma cells (A-172), Human primary umbilical vein endothelial cells	Extrusion bioprinter	Gelatin methacryloyl, Alginate, Fibrinogen	Photo-Induced Cross-Linking	Live/dead assay kit and Fluorescence microscopy	24
3D bioprinted Glioblastoma modelling	Glioma stem cell line (SU3), Mesenchymal stem cells	Coaxial Extrusion bioprinter, Extrusion bioprinter	Sodium alginate, Gelatin, Fibrinogen	$CaCl_2$, Thrombin	Live/dead assay kit, Fluorescence microscopy	25

Table S2-S2. Bioprinting Parameters for Tumor Models with Neuroblastoma Cells.

Application	Cell type	Bioprinting technique	Bioink type	Crosslink Method	Viability Technique	Reference
3D bioprinted neural tissue modelling	Neuroblastoma cells (SH-SY5Y), Induced pluripotent stem cells, Neural stem cells	Extrusion bioprinter	Sodium alginate, Gelatin	$CaCl_2$	Live/dead viability assay kit, Confocal microscopy	26
3D bioprinted Neuroblastoma modelling	Neuroblastoma cells (SH-SY5Y)	Multi-nozzle extrusion bioprinter	Sodium alginate, Gelatin	Transglutaminase, $CaCl_2$	Live/dead viability assay kit, Confocal microscopy	27
3D bioprinted neural tissue modelling	Neuroblastoma cells (SH-SY5Y), Induced pluripotent stem cells	Extrusion bioprinter	Hyaluronic acid, Poly (ethylene glycol)	Azide-alkyne cycloaddition	Alamar blue viability assay, Confocal microscopy	28
3D bioprinted astrocyte model	Neuroblastoma cells (SH-SY5Y), Human fetal primary astrocytes, Glioblastoma cells (U87)	Extrusion bioprinter	Hyaluronic acid, RGD, IKVAV peptides	Azide–alkyne cycloaddition	Alamar blue viability assay	29
3D bioprinted Neuroblastoma modelling	Neuroblastoma cells (SK-N-BE (2))	Extrusion bioprinter	Methacrylated alginate, Gelatin methacryloyl	Photo-Induced Cross-Linking	Optical microscopy	30

Table S2-S2. Bioprinting Parameters for Tumor Models with Neuroblastoma Cells (Cont.).

Application	Cell type	Bioprinting technique	Bioink type	Crosslink Method	Viability Technique	Reference
3D bioprinted Neuroblastoma modelling	Neuroblastoma cells (IMR-32)	Micro extrusion bioprinter	Gelatin, Chitosan	Transglutaminase	Live/dead viability assay kit, Two color flow cytometry	31
3D bioprinted Neuroblastoma modelling	Neuroblastoma cells (SK-N-BE (2)), NB cells (SH-SY5Y), SW10 mouse Schwann cells	Extrusion bioprinter	Methacrylated alginate, Gelatin methacryloyl	Photo-Induced Cross-Linking	Optical microscopy	32
3D bioprinted Neuroblastoma modelling	Neuroblastoma cells (SK-N-BE (2)), NB cells (SH-SY5Y), SW10 mouse Schwann cells	Extrusion bioprinter	Methacrylated alginate, Gelatin methacryloyl	Photo-Induced Cross-Linking		33
3D bioprinted Neuroblastoma modelling	Umbilical cord-derived mesenchymal stromal cells, Human primary umbilical vein endothelial cells, Neuroblastoma cells (SH-SY5Y)	Droplet based bioprinter	Type I collagen, Agarose-type I		Two photon laser scanning microscope	34

Table S2-S2. Bioprinting Parameters for Tumor Models with Neuroblastoma Cells (Cont.)

Application	Cell type	Bioprinting technique	Bioink type	Crosslink Method	Viability Technique	Reference
3D bioprinted Neuroblastoma vascular modelling	Patient derived Neuroblastoma cells (IMR5), Human primary umbilical vein endothelial cells	Extrusion bioprinter (Embedded bioprinting method)	Gelatin methacryloyl, Carbopol as supporting bath material	Photo-Induced Cross-Linking	Live/dead viability assay kit, Confocal microscopy and Alamar blue viability assay	35
3D bioprinted Neuroblastoma modelling	Neuroblastoma cells (IMR-32), Human embryonic kidney 293 cells (HEK293, CRL-1573)	Multi nozzle extrusion bioprinter	Collagen, Sodium alginate, Gelatin	$CaCl_2$	XTT assay	36
3D bioprinted Neuroblastoma modelling	Neuroblastoma cells (SK-N-AS, SK-N-BE (2)), Patient derived Neuroblastoma cells (COA3, COA6), gastroenteropancreatic human cells (NETPDX), Neuroendocrine-like tumor cells (COA109)	Extrusion bioprinter	Sodium alginate, Gelatin	$CaCl_2$	Cell Titer-Glo 3D	37

Table S2-S2. Bioprinting Parameters for Tumor Models with Neuroblastoma Cells (Cont.)

Application	Cell type	Bioprinting technique	Bioink type	Crosslink Method	Viability Technique	Reference
3D organ on chips Neuroblastoma vascular modeling	Human primary umbilical vein endothelial cells, Neuroblastoma cells (STANB15), Human fibroblast cells (PCS-201-010), Primary adipose-tissue derived stem cells, Mesenchymal like stem cells differentiated from induced pluripotent stem cells	Extrusion bioprinter, Droplet based bioprinter	Pluronic F127, Gelatin methacryloyl, Fibrinogen	Photo-Induced Cross-Linking and Thrombin	Alamar blue viability assay, Confocal microscopy	38
3D bioprinted Neuroblastoma modelling	Neuroblastoma cells (SK-N-BE)	Extrusion bioprinter (Embedded bioprinting method)	Cellulose, Sodium alginate, Gelatin as a supporting bath material	$CaCl_2$	Live/dead assay kit, Fluorescent microscopy	39
3D bioprinted neural tissue modeling	Neuro2a mouse Neuroblastoma cells	Extrusion bioprinter	N, O-Carboxymethyl Chitosan, Agarose	$CaCl_2$	Live/dead assay kit, Confocal microscope	40

REFERENCES

(1) Heinrich, M. A.; Bansal, R.; Lammers, T.; Zhang, Y. S.; Michel Schiffelers, R.; Prakash, J. 3D-Bioprinted Mini-Brain: A Glioblastoma Model to Study Cellular Interactions and Therapeutics. *Advanced Materials* **2019**, *31* (14), 1806590. https://doi.org/10.1002/adma.201806590.

(2) Lee, V. K.; Dai, G.; Zou, H.; Yoo, S.-S. Generation of 3-D Glioblastoma-Vascular Niche Using 3-D Bioprinting. In *Proceedings of the 2015 41st Annual Northeast Biomedical Engineering Conference (NEBC), Troy, NY, USA, 17–19 April 2015*; IEEE: Piscataway, NJ, USA, 2015; pp 1–2. https://doi.org/10.1109/NEBEC.2015.7117111.

(3) Han, S.; Kim, S.; Chen, Z.; Shin, H. K.; Lee, S. Y.; Moon, H. E.; Paek, S. H.; Park, S. 3D Bioprinted Vascularized Tumour for Drug Testing. *Int J Mol Sci* **2020**, *21* (8), 2993. https://doi.org/10.3390/ijms21082993.

(4) van Pel, D. M.; Harada, K.; Song, D.; Naus, C. C.; Sin, W. C. Modelling Glioma Invasion Using 3D Bioprinting and Scaffold-Free 3D Culture. *J Cell Commun Signal* **2018**, *12* (4), 723–730. https://doi.org/10.1007/s12079-018-0469-z.

(5) Lee, C.; Abelseth, E.; de la Vega, L.; Willerth, S. M. Bioprinting a Novel Glioblastoma Tumor Model Using a Fibrin-Based Bioink for Drug Screening. *Mater Today Chem* **2019**, *12*, 78–84. https://doi.org/10.1016/j.mtchem.2018.12.005.

(6) Ozturk, M. S.; Lee, V. K.; Zou, H.; Friedel, R. H.; Intes, X.; Dai, G. High-Resolution Tomographic Analysis of in Vitro 3D Glioblastoma Tumor Model under Long-Term Drug Treatment. *Sci. Adv* **2020**, *6*, 7513–7519. https://doi.org/10.1002/smll.202006050.

(7) Hermida, M. A.; Kumar, J. D.; Schwarz, D.; Laverty, K. G.; Di Bartolo, A.; Ardron, M.; Bogomolnijs, M.; Clavreul, A.; Brennan, P. M.; Wiegand, U. K.; Melchels, F. P.; Shu, W.; Leslie, N. R. Three Dimensional in Vitro Models of Cancer: Bioprinting Multilineage Glioblastoma Models. *Adv Biol Regul* **2020**, *75*, 100658. https://doi.org/10.1016/j.jbior.2019.100658.

(8) Dai, X.; Ma, C.; Lan, Q.; Xu, T. 3D Bioprinted Glioma Stem Cells for Brain Tumor Model and Applications of Drug Susceptibility. *Biofabrication* **2016**, *8* (4), 045005. https://doi.org/10.1088/1758-5090/8/4/045005.

(9) Dai, X.; Liu, L.; Ouyang, J.; Li, X.; Zhang, X.; Lan, Q.; Xu, T. Coaxial 3D Bioprinting of Self-Assembled Multicellular Heterogeneous Tumor Fibers. *Sci Rep* **2017**, *7* (1), 1457. https://doi.org/10.1038/s41598-017-01581-y.

(10) Wang, X.; Li, X.; Dai, X.; Zhang, X.; Zhang, J.; Xu, T.; Lan, Q. Coaxial Extrusion Bioprinted Shell-Core Hydrogel Microfibers Mimic Glioma Microenvironment and Enhance the Drug Resistance of Cancer Cells. *Colloids Surf B Biointerfaces* **2018**, *171*, 291–299. https://doi.org/10.1016/j.colsurfb.2018.07.042.

(11) Wang, X.; Li, X.; Dai, X.; Zhang, X.; Zhang, J.; Xu, T.; Lan, Q. Bioprinting of Glioma Stem Cells Improves Their Endotheliogenic Potential. *Colloids Surf B Biointerfaces* **2018**, *171*, 629–637. https://doi.org/10.1016/j.colsurfb.2018.08.006.

(12) Wang, X.; Dai, X.; Zhang, X.; Ma, C.; Li, X.; Xu, T.; Lan, Q. 3D Bioprinted Glioma Cell-Laden Scaffolds Enriching Glioma Stem Cells via Epithelial–Mesenchymal Transition. *J Biomed Mater Res A* **2019**, *107* (2), 383–391. https://doi.org/10.1002/jbm.a.36549.

(13) Smits, I. P. M.; Blaschuk, O. W.; Willerth, S. M. Novel N-Cadherin Antagonist Causes Glioblastoma Cell Death in a 3D Bioprinted Co-Culture Model. *Biochem Biophys Res Commun* **2020**, *529* (2), 162–168. https://doi.org/10.1016/j.bbrc.2020.06.001.

(14) Yi, H. G.; Jeong, Y. H.; Kim, Y.; Choi, Y. J.; Moon, H. E.; Park, S. H.; Kang, K. S.; Bae, M.; Jang, J.; Youn, H.; Paek, S. H.; Cho, D. W. A Bioprinted Human-Glioblastoma-on-a-Chip for the Identification of Patient-Specific Responses to Chemoradiotherapy. *Nat Biomed Eng* **2019**, *3* (7), 509–519. https://doi.org/10.1038/s41551-019-0363-x.

(15) Tang, M.; Tiwari, S. K.; Agrawal, K.; Tan, M.; Dang, J.; Tam, T.; Tian, J.; Wan, X.; Schimelman, J.; You, S.; Xia, Q.; Rana, T. M.; Chen, S. Rapid 3D Bioprinting of Glioblastoma Model Mimicking Native Biophysical Heterogeneity. *Small* **2021**, *17* (15), e2006050. https://doi.org/10.1002/smll.202006050.

(16) Tang, M.; Xie, Q.; Gimple, R. C.; Zhong, Z.; Tam, T.; Tian, J.; Kidwell, R. L.; Wu, Q.; Prager, B. C.; Qiu, Z.; Yu, A.; Zhu, Z.; Mesci, P.; Jing, H.; Schimelman, J.; Wang, P.; Lee, D.; Lorenzini, M. H.; Dixit, D.; Zhao, L.; Bhargava, S.; Miller, T. E.; Wan, X.; Tang, J.; Sun, B.; Cravatt, B. F.; Muotri, A. R.; Chen, S.; Rich, J. N. Three-Dimensional Bioprinted Glioblastoma Microenvironments Model Cellular Dependencies and Immune Interactions. *Cell Res* **2020**, *30* (10), 833–853. https://doi.org/10.1038/s41422-020-0338-1.

(17) Zhang, J.; Chen, F.; He, Z.; Ma, Y.; Uchiyama, K.; Lin, J. M. A Novel Approach for Precisely Controlled Multiple Cell Patterning in Microfluidic Chips by Inkjet Printing and the Detection of Drug Metabolism and Diffusion. *Analyst* **2016**, *141* (10), 2940–2947. https://doi.org/10.1039/c6an00395h.

(18) Dusserre, N.; Stachowicz, M. L.; Medina, C.; Henri, B.; Fricain, J. C.; Paris, F.; Oliveira, H. Microvalve Bioprinting as a Biofabrication Tool to Decipher Tumor and Endothelial Cell Crosstalk: Application to a Simplified Glioblastoma Model. *Bioprinting* **2021**, *24*, e00178. https://doi.org/10.1016/j.bprint.2021.e00178.

(19) Neufeld, L.; Yeini, E.; Reisman, N.; Shtilerman, Y.; Ben-Shushan, D.; Pozzi, S.; Madi, A.; Tiram, G.; Eldar-Boock, A.; Ferber, S.; Grossman, R.; Ram, Z.; Satchi-Fainaro, R. Microengineered Perfusable 3D-Bioprinted Glioblastoma Model for in Vivo Mimicry of Tumor Microenvironment. *Sci. Adv* **2021**, *7* (34), 9119–9137.

(20) Wang, X.; Li, X.; Zhang, Y.; Long, X.; Zhang, H.; Xu, T.; Niu, C. Coaxially Bioprinted Cell-Laden Tubular-Like Structure for Studying Glioma Angiogenesis. *Front Bioeng Biotechnol* **2021**, *9*, 761861. https://doi.org/10.3389/fbioe.2021.761861.

(21) Wang, X.; Li, X.; Ding, J.; Long, X.; Zhang, H.; Zhang, X.; Jiang, X.; Xu, T. 3D Bioprinted Glioma Microenvironment for Glioma Vascularization. *J Biomed Mater Res A* **2021**, *109* (6), 915–925. https://doi.org/10.1002/jbm.a.37082.

(22) Maloney, E.; Clark, C.; Sivakumar, H.; Yoo, K.; Aleman, J.; Rajan, S. A. P.; Forsythe, S.; Mazzocchi, A.; Laxton, A. W.; Tatter, S. B.; Strowd, R. E.; Votanopoulos, K. I.; Skardal, A. Immersion Bioprinting of Tumor Organoids in Multi-Well Plates for Increasing Chemotherapy Screening Throughput. *Micromachines (Basel)* **2020**, *11* (2), 208. https://doi.org/10.3390/mi11020208.

(23) Schwartz, R. L. Bioprinted in Vitro Model of Human Glioblastoma. M.S. thesis, Rowan University, Glassboro, NJ, USA, 2020. https://rdw.rowan.edu/etd/?utm_source=rdw.rowan.edu%2Fetd%2F2837&utm_medium=PDF&utm_campaign=PDFCoverPages (accessed 2023-09-30).

(24) Silvani, G.; Basirun, C.; Wu, H.; Mehner, C.; Poole, K.; Bradbury, P.; Chou, J. A 3D-Bioprinted Vascularized Glioblastoma-on-a-Chip for Studying the Impact of Simulated Microgravity as a Novel Pre-Clinical Approach in Brain Tumor Therapy. *Adv Ther (Weinh)* **2021**, *4* (11), 2100106. https://doi.org/10.1002/adtp.202100106.

(25) Dai, X.; Shao, Y.; Tian, X.; Cao, X.; Ye, L.; Gao, P.; Cheng, H.; Wang, X. Fusion between Glioma Stem Cells and Mesenchymal Stem Cells Promotes Malignant Progression in 3D-Bioprinted Models. *ACS Appl Mater Interfaces* **2022**, *14* (31), 35344–35356. https://doi.org/10.1021/acsami.2c06658.

(26) Fantini, V.; Bordoni, M.; Scocozza, F.; Conti, M.; Scarian, E.; Carelli, S.; Di Giulio, A. M.; Marconi, S.; Pansarasa, O.; Auricchio, F.; Cereda, C. Bioink Composition and Printing Parameters for 3D Modeling Neural Tissue. *Cells* **2019**, *8* (8), 830. https://doi.org/10.3390/cells8080830.

(27) Chen, Q.; Tian, X.; Fan, J.; Tong, H.; Ao, Q.; Wang, X. An Interpenetrating Alginate/Gelatin Network for Three-Dimensional (3D) Cell Cultures and Organ Bioprinting. *Molecules* **2020**, *25* (3), 756. https://doi.org/10.3390/molecules25030756.

(28) Jury, M.; Matthiesen, I.; Rasti Boroojeni, F.; Ludwig, S. L.; Civitelli, L.; Winkler, T. E.; Selegård, R.; Herland, A.; Aili, D. Bioorthogonally Cross-Linked Hyaluronan–Laminin Hydrogels for 3D Neuronal Cell Culture and Biofabrication. *Adv Healthc Mater* **2022**, *11* (11), 2102097. https://doi.org/10.1002/adhm.202102097.

(29) Matthiesen, I.; Jury, M.; Rasti Boroojeni, F.; Ludwig, S. L.; Holzreuter, M.; Buchmann, S.; Åman Träger, A.; Selegård, R.; Winkler, T. E.; Aili, D.; Herland, A. Astrocyte 3D Culture and Bioprinting Using Peptide Functionalized Hyaluronan Hydrogels. *Sci Technol Adv Mater* **2023**, *24* (1), 2165871. https://doi.org/10.1080/14686996.2023.2165871.

(30) Monferrer, E.; Martín-Vañó, S.; Carretero, A.; García-Lizarribar, A.; Burgos-Panadero, R.; Navarro, S.; Samitier, J.; Noguera, R. A Three-Dimensional Bioprinted Model to Evaluate the Effect of Stiffness on Neuroblastoma Cell Cluster Dynamics and Behavior. *Sci Rep* **2020**, *10* (1), 6370. https://doi.org/10.1038/s41598-020-62986-w.

(31) Roehm, K. D.; Madihally, S. V. Bioprinted Chitosan-Gelatin Thermosensitive Hydrogels Using an Inexpensive 3D Printer. *Biofabrication* **2018**, *10* (1), 015002. https://doi.org/10.1088/1758-5090/aa96dd.

(32) Monferrer, E.; Sanegre, S.; Martín-Vañó, S.; García-Lizarribar, A.; Burgos-Panadero, R.; López-Carrasco, A.; Navarro, S.; Samitier, J.; Noguera, R. Digital Image Analysis Applied to Tumor Cell Proliferation, Aggressiveness, and Migration-Related Protein Synthesis in Neuroblastoma 3d Models. *Int J Mol Sci* **2020**, *21* (22), 1–17. https://doi.org/10.3390/ijms21228676.

(33) López-Carrasco, A.; Martín-Vañó, S.; Burgos-Panadero, R.; Monferrer, E.; Berbegall, A. P.; Fernández-Blanco, B.; Navarro, S.; Noguera, R. Impact of Extracellular Matrix Stiffness on Genomic Heterogeneity in MYCN-Amplified Neuroblastoma Cell Line. *Journal of Experimental and Clinical Cancer Research* **2020**, *39* (1), 1–13. https://doi.org/10.1186/s13046-020-01729-1.

(34) Campos, D. F. D.; Marquez, A. B.; O'seanain, C.; Fischer, H.; Blaeser, A.; Vogt, M.; Corallo, D.; Aveic, S. Exploring Cancer Cell Behavior in Vitro in Three-Dimensional Multicellular Bioprintable Collagen-Based Hydrogels. *Cancers (Basel)* **2019**, *11* (2), 180. https://doi.org/10.3390/cancers11020180.

(35) Ning, L.; Shim, J.; Tomov, M. L.; Liu, R.; Mehta, R.; Mingee, A.; Hwang, B.; Jin, L.; Mantalaris, A.; Xu, C.; Mahmoudi, M.; Goldsmith, K. C.; Serpooshan, V. A 3D Bioprinted in Vitro Model of Neuroblastoma Recapitulates Dynamic Tumor-Endothelial Cell Interactions Contributing to Solid Tumor Aggressive Behavior. *Advanced Science* **2022**, *9* (23), 2200244. https://doi.org/10.1002/advs.202200244.

(36) Wu, D.; Berg, J.; Arlt, B.; Röhrs, V.; Al-Zeer, M. A.; Deubzer, H. E.; Kurreck, J. Bioprinted Cancer Model of Neuroblastoma in a Renal Microenvironment as an Efficiently Applicable

Drug Testing Platform. *Int J Mol Sci* **2022**, *23* (1), 122. https://doi.org/10.3390/ijms23010122.

(37) Quinn, C. H.; Beierle, A. M.; Julson, J. R.; Erwin, M. E.; Alrefai, H.; Markert, H. R.; Stewart, J. E.; Claire Hutchins, S.; Bownes, L. V; Aye, J. M.; Mroczek-Musulman, E.; Hicks, P. H.; Yoon, K. J.; Willey, C. D.; Beierle, E. A. International Journal of Bioprinting Using 3D-Bioprinted Models to Study Pediatric Neural Crest-Derived Tumors International Journal of Bioprinting 3D Bioprinted Models in Pediatric Tumors. **2023**, *9* (4), 723. https://doi.org/10.18063/ijb.723.

(38) Nothdurfter, D.; Ploner, C.; Coraça-Huber, D. C.; Wilflingseder, D.; Müller, T.; Hermann, M.; Hagenbuchner, J.; Ausserlechner, M. J. 3D Bioprinted, Vascularized Neuroblastoma Tumor Environment in Fluidic Chip Devices for Precision Medicine Drug Testing. *Biofabrication* **2022**, *14* (3), 035002. https://doi.org/10.1088/1758-5090/ac5fb7.

(39) Lewicki, J.; Bergman, J.; Kerins, C.; Hermanson, O. Optimization of 3D Bioprinting of Human Neuroblastoma Cells Using Sodium Alginate Hydrogel. *Bioprinting* **2019**, *16*, e00053. https://doi.org/10.1016/j.bprint.2019.e00053.

(40) Butler, H. M.; Naseri, E.; MacDonald, D. S.; Tasker, R. A.; Ahmadi, A. Investigation of Rheology, Printability, and Biocompatibility of N,O-Carboxymethyl Chitosan and Agarose Bioinks for 3D Bioprinting of Neuron Cells. *Materialia (Oxf)* **2021**, *18*, 101169. https://doi.org/10.1016/j.mtla.2021.101169.
